# Accurate *ab initio* gene prediction in eukaryotes with Tiberius in multiple clades

**DOI:** 10.64898/2026.04.24.720536

**Published:** 2026-04-28

**Authors:** Lars Gabriel, Tomáš Brůna, Asees Kaur, Anish Krishnan, Felix Ortmann, Asaf Salamov, Samuel Talbot, Felix Becker, Richard Krieg, Christopher W. Wheat, Igor V. Grigoriev, Mario Stanke, Katharina J. Hoff

**Affiliations:** 1 Institute of Mathematics and Computer Science, University of Greifswald, Walther-Rathenau-Str. 47, 17489 Greifswald, MV, Germany; 2 DOE Joint Genome Institute, Lawrence Berkeley National Laboratory, 1 Cyclotron Road, Berkeley, 94720, California, USA; 3 University of California Merced, Merced, CA 95343, USA; 4 Department of Plant and Microbial Biology, University of California Berkeley, Berkeley, CA 94720, USA; 5 Center for Quantitative Life Sciences, Oregon State University, OR 97331, Oregon, USA; 6 Department of Zoology, Stockholm University, Svante Arrhenius väg 18b, SE-106 91, Stockholm, Sweden

**Keywords:** gene prediction, genome annotation, deep learning, hidden Markov model, eukaryotes

## Abstract

Eukaryotic genome annotation is currently bottlenecked by limitations in the generality, scalability and accuracy of computational methods. Deep learning approaches have recently achieved large improvements in *ab initio* gene prediction accuracy. We extend the deep learning-based *ab initio* gene predictor Tiberius beyond mammals by training lineage-specific models for Mesangiospermae, Fungi, Vertebrata, Insecta, Chlorophyta and Bacillariophyta. Across a benchmark of 33 species, Tiberius consistently achieves higher accuracy than the other evaluated *ab initio* methods, Helixer and ANNEVO, while also having the fastest runtimes overall. Compared with BRAKER3, which incorporates RNA-Seq and protein evidence, Tiberius approaches state-of-the-art accuracy in Mesangiospermae, Fungi, Bacillariophyta and Chlorophyta, while being on average 80 times faster when using a GPU.

**Availability and implementation:**

https://github.com/Gaius-Augustus/Tiberius

## 1 Introduction

Accurate identification of protein-coding genes is a critical step in genome annotation, yet fewer than 20% of the currently available genomes at NCBI Datasets [O’Leary et al., 2024] have associated gene annotations. This problem may amplify quickly as the scale of genome sequencing continues to expand through initiatives such as the Earth BioGenome Project [Blaxter et al., 2025]. Improving accuracy, ease of application and compute efficiency is therefore essential to homogeneously annotate the growing wealth of available genomes.

While evidence-based annotation methods that integrate RNA-Seq data and protein homology, such as BRAKER3 [Gabriel et al., 2024b], achieve high accuracy, they require substantial extrinsic evidence that may not be available for many newly sequenced organisms. In addition, BRAKER3’s computational requirements can be prohibitive when annotating large numbers of genomes; this is exacerbated by the need for generating species-specific repeat libraries for rigorous repeat masking prior to gene annotation.

Until recently, *ab initio* gene prediction methods, which identify genes solely from the genome sequence, have severely lagged behind evidence-based approaches in terms of accuracy. Additionally, many *ab initio* gene predictors, such as AUGUSTUS [Stanke et al., 2003], require species-specific training, which is time-consuming and requires extrinsic evidence such as RNA-Seq data or homologous proteins to achieve decent accuracy.

Helixer [Holst et al., 2025], introduced in 2023, showed that combining deep learning layers and hidden Markov model (HMM) postprocessing could improve over the “shallow” HMM gene-finder AUGUSTUS. Building on the findings of the Helixer project, we recently introduced Tiberius [Gabriel et al., 2024a], an end-to-end deep learning *ab initio* gene predictor that achieves high accuracy for mammalian genomes, approaching the accuracy of BRAKER3 despite using no extrinsic evidence. More recently, ANNEVO has been introduced as another deep learning-based *ab initio* gene predictor that outcompetes Helixer in terms of accuracy [Zhang et al., 2026].

The original Tiberius was trained only on mammals, limiting its applicability across eukaryotes. Here, we extend Tiberius to six additional clades: Mesangiospermae, Fungi, Vertebrata, Insecta, Chlorophyta and Bacillariophyta. We show that these models outperform Helixer and ANNEVO across almost all benchmarks while approaching BRAKER3 accuracy in several lineages.

## 2 Materials and methods

### 2.1 Model training

Clade-specific Tiberius models were trained for Mesangiospermae, Fungi, Vertebrata, Insecta, Chlorophyta and Bacillariophyta. All models were trained on unmasked genomes (training, validation, test data see Suppl. Tables 1–9) following the procedure described in the original Tiberius publication (see Suppl. Methods). Details on hardware and model training are provided in Suppl. Tables 30–31. The overall architecture remained unchanged, except for adjusted layer widths in Insecta and Vertebrata (Suppl. Table 31).

### 2.2 Implementation improvements

The Python backend of Tiberius was mostly reimplemented from scratch and modularized into two separate packages. The first package, *bricks2marble*, provides data handling and annotation logic. The second package, *hidten*, implements the differentiable HMM layer. In addition, the new implementation is compatible with more recent TensorFlow versions (>2.13), making it easier to install and maintain.

### 2.3 Benchmark comparison

We benchmarked Tiberius against the deep learning *ab initio* gene prediction tools, Helixer and ANNEVO. For each tool, clade-appropriate pretrained models provided by the respective authors were used where available (Suppl. Table 29). In addition, we compared performance with that of BRAKER3 as a representative state-of-the-art general-purpose gene prediction pipeline. In contrast to the *ab initio* tools, BRAKER3 requires transcriptomic and protein evidence. RNA-Seq input was generated using VARUS [Stanke et al., 2019]. As protein evidence, we used the combined proteomes of all Tiberius training species within the respective target clade.

### 2.4 Evaluation metrics

Prediction accuracy was evaluated using standard metrics for gene prediction. Predicted gene structures were compared to reference annotations at the coding sequence (CDS) exon and gene level, with untranslated regions (UTRs) excluded from the evaluation. A predicted instance was considered a true positive only if its structure exactly matched a reference instance. At the gene level, a locus was counted as correctly predicted if at least one transcript matched the reference annotation exactly. Sensitivity, precision and F1 score were calculated from the resulting counts using gffcompare v0.12.10 [Pertea and Pertea, 2020].

## 3 Results

### 3.1 Accuracy comparison with other *ab initio* methods

**Figure 1:**
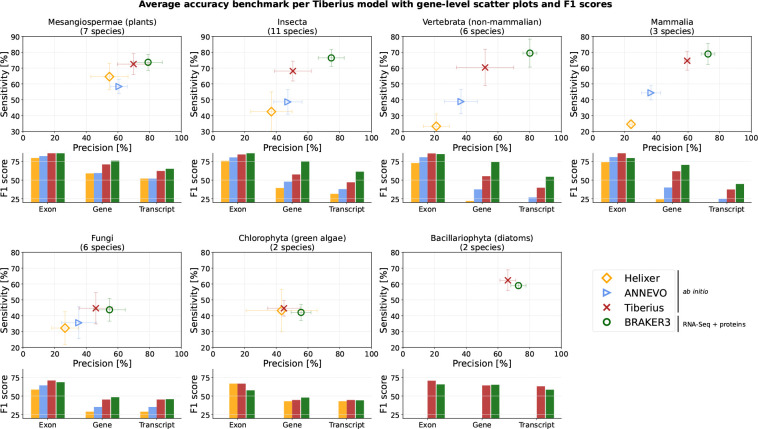
Average accuracy across test species for each Tiberius model, compared with *ab initio* predictions from Helixer and ANNEVO and evidence-supported predictions from BRAKER3. Gene-level sensitivity and precision are shown as scatter plots; CDS-exon-, transcript- and gene-level F1 scores are shown as bar plots.

On all five clades (33 test species) for which all three tools were trained, Tiberius achieved higher exon-, gene- and transcript-level accuracy than Helixer and ANNEVO (Figure 1). At gene level, Tiberius improved F1 scores over Helixer on average by 12–37 percentage points per clade, with the largest differences in Mammalia and non-mammalian Verte-brata (Suppl. Tables 19–26). Compared with ANNEVO, Tiberius achieved a 10–21 percentage points higher gene-level F1 score, with higher differences in sensitivity than precision. Tiberius’ exon-level F1 score was also consistently higher, by about 3 percentage points on average. Across the two Chlorophyta test species, Helixer achieved the higher gene-level F1 score on *B. prasinos* (55.8% vs. 50.1%), whereas Tiberius performed better on *E. debaryana* (39.3% vs. 30.3%). ANNEVO does not provide a pretrained model for Chlorophyta. Of the 33 test species, Helixer included 17 in its training or validation data, whereas ANNEVO included two and Tiberius included none (Suppl. Table 18). Tiberius offers the first deep learning gene prediction model for the protists Bacillariophyta. Therefore, on this clade, only comparisons to BRAKER3 were conducted.

### 3.2 Accuracy comparison with BRAKER3

BRAKER3, which additionally uses RNA-Seq and protein evidence, achieved higher overall gene- and transcript-level accuracy than Tiberius, with overall F1 score differences of about 12 and nine percentage points, respectively. The largest gap was in non-mammalian Vertebrata, where BRAKER3 exceeded Tiberius by about 19 percentage points at gene level and 15 at transcript level F1 scores. In Mesangiospermae, Fungi, Bacillariophyta and Chlorophyta, transcript-level accuracy was similar and in Bacillariophyta, Tiberius exceeded BRAKER3 by five percentage points in F1 score. For these four groups, gene-level F1 scores differed by less than three percentage points. At exon level, both methods performed similarly, with Tiberius having on average an ≤ 1 percentage point higher F1 score.

### 3.3 Runtime comparison

A direct runtime comparison between BRAKER3 and the deep learning methods is limited because only the latter used a GPU. All tools used 72 threads of an AMD EPYC 7773X 64-Core Processor and the deep learning methods additionally used an NVIDIA A100-SXM4–80GB GPU. Across the 33 species benchmarked with all four tools, average runtimes were 26 min for Tiberius, 30 min for ANNEVO, 178 min for Helixer, and 2170 min for BRAKER3 (Supplementary Tables 10–17). BRAKER3 was particularly slow on large genomes; in non-mammalian Vertebrata it required on average 3035 min per genome, whereas Tiberius and ANNEVO remained below 60 min. Among the *ab initio* methods, Tiberius and ANNEVO were the fastest and about six times faster than Helixer on average.

### 3.4 Effect of implementation changes

Implementation improvements to Tiberius substantially reduced runtime while leaving prediction accuracy nearly unchanged. Across all 37 test species, the average runtime decreased from 35 min to 24 min per genome, a reduction of 31.4% (Suppl. Table 28). Prediction accuracy remained nearly unchanged between the two versions, with average F1 score differences of at most 0.3 percentage points (Suppl. Table 27).

### 3.5 Large-scale vertebrate annotation

Tiberius has also been applied beyond the benchmark setting to annotate 2,948 vertebrate assemblies totaling nearly 6 trillion base pairs (5,987,909,637,336 bp) that are available at https://bioinf.uni-greifswald.de/bioinf/tiberius/genes/tib-tbl-vert.html (362 of primates, 1120 of other mammals, 497 of birds, 650 of fishes, 319 of other vertebrates).

## 4 Discussion

### 4.1 Comparison with other *ab initio* methods

Our results show that Tiberius is transferable beyond Mammalia, and its models are now applicable to 92% of currently available eukaryotic assemblies (see Suppl. Figure 1). Across this phylogenetic range, Tiberius was consistently more accurate than the other evaluated *ab initio* methods. Interpreting these comparisons is complicated by differences in training-data composition. Helixer, and to a lesser extent ANNEVO, included test species we selected for benchmarking in their training sets, giving them an advantage on these species [Pagès-Gallego and De Ridder, 2023].

For our models, training data were assembled largely through collaboration with clade experts. The fungal comparison with ANNEVO is the cleanest in methodological respect, as both methods were trained on the same dataset. Under this condition, Tiberius still outperformed ANNEVO, although the gap was smaller than in several other clades.

### 4.2 Model training

The variation in accuracy between clades suggests that reference annotation availability and quality remain major limiting factors. This is particularly evident for Chlorophyta, where training data are sparse and annotations are more heterogeneous than in better curated clades. For Insecta and Bacillariophyta, we supplemented publicly available annotations with BRAKER annotations generated by our group to improve clade coverage [Saenko et al., 2026, Nenasheva et al., 2025]. In Fungi, lower accuracy likely reflects broader phylogenetic diversity and heterogeneous annotations rather than insufficient training data. Further improvements will therefore require more consistently curated reference annotations across underrepresented clades.

### 4.3 Accuracy metrics

We evaluated predictions using exact CDS-exon-, transcript- and gene-level sensitivity, precision and F1 score, excluding UTRs because Tiberius does not predict them. These structure-level metrics have been traditionally used for benchmarking and remain the standard for gene prediction because they require correct reconstruction of exon boundaries and complete gene models [Burset and Guigo, 1996, Parra et al., 2003]. Other measures, such as BUSCO completeness [Simão et al., 2015], nucleotide-level accuracy and protein-level similarity remain useful, particularly as a quality control measure for newly annotated genomes, but are not equivalent to exact structure-level assessment [Gabriel et al., 2024a]. These measures can miss biologically relevant errors in exon chaining and gene structure, which can affect downstream analyses [Kirilenko et al., 2023, Meyer et al., 2020].

### 4.4 Comparison with BRAKER3

The comparison with BRAKER3 indicates that Tiberius’ main limitation is not CDS-exon-boundary detection, but the reconstruction of complete gene structures in complex genomes. This is supported by near parity of both methods at exon level, while a substantial gap remains at gene level in Vertebrata.

A limitation of the deep-learning *ab initio* methods evaluated here is that they predict only a single isoform per locus. This constrains transcript-level accuracy and gives BRAKER3 a systematic advantage at gene level, since a locus is counted as correct when at least one predicted isoform matches the reference. Extending deep-learning gene-finders to support multi-isoform prediction would improve biological realism. Another natural route to narrow the gap is the integration of extrinsic evidence, although this can come at substantial computational cost, as BRAKER3 required over 80 times more compute time than Tiberius.

## 5 Conclusion

We extend available Tiberius models from Mammalia to six additional clades, making Tiberius applicable to the vast majority of publicly available assemblies. Tiberius consistently outperforms the other evaluated deep learning *ab initio* methods and, in several clades, approaches the accuracy of BRAKER3 while requiring far less runtime. These results make Tiberius a practical choice for highly accurate large-scale genome annotation, particularly when extrinsic evidence is unavailable or annotation throughput is limiting.

## Supplementary Material

Supplement 1
