# Supplementary material for: Accurate ab initio gene prediction in eukaryotes with Tiberius in multiple clades
Source: bioRxiv. 2026 Apr 28:2026.04.24.720536. Preprint. [Version 1] doi: 10.64898/2026.04.24.720536 (PMC13142403; doi:10.64898/2026.04.24.720536)
Supplement: Supplement 1 [file media-1.pdf]

Accurate *ab initio* gene prediction in eukaryotes with Tiberius in multiple clades

Supplementary Materials

Lars Gabriel[1], Tomáš Brůna[2], Asees Kaur[3], Anish Krishnan[4], Felix Ortmann[1], Asaf Salamov[2], Samuel Talbot[5], Felix Becker[1], Richard Krieg[1], Christopher W. Wheat[6], Igor V. Grigoriev[2,4], Mario Stanke[1,*], and Katharina J. Hoff[1,*]

[1]Institute of Mathematics and Computer Science, University of Greifswald, Walther-Rathenau-Str. 47, 17489 Greifswald, Germany
[2]DOE Joint Genome Institute, Lawrence Berkeley National Laboratory, 1 Cyclotron Road, Berkeley, 94720, California, USA
[3]University of California Merced, Merced, CA 95343, USA
[4]Department of Plant and Microbial Biology, University of California Berkeley, Berkeley, CA 94720, USA
[5]Center for Quantitative Life Sciences, Oregon State University, OR 97331, USA
[6]Department of Zoology, Stockholm University, Sweden
[*]Corresponding authors: mario.stanke@uni-greifswald.de, katharina.hoff@uni-greifswald.de

Supplementary Figures

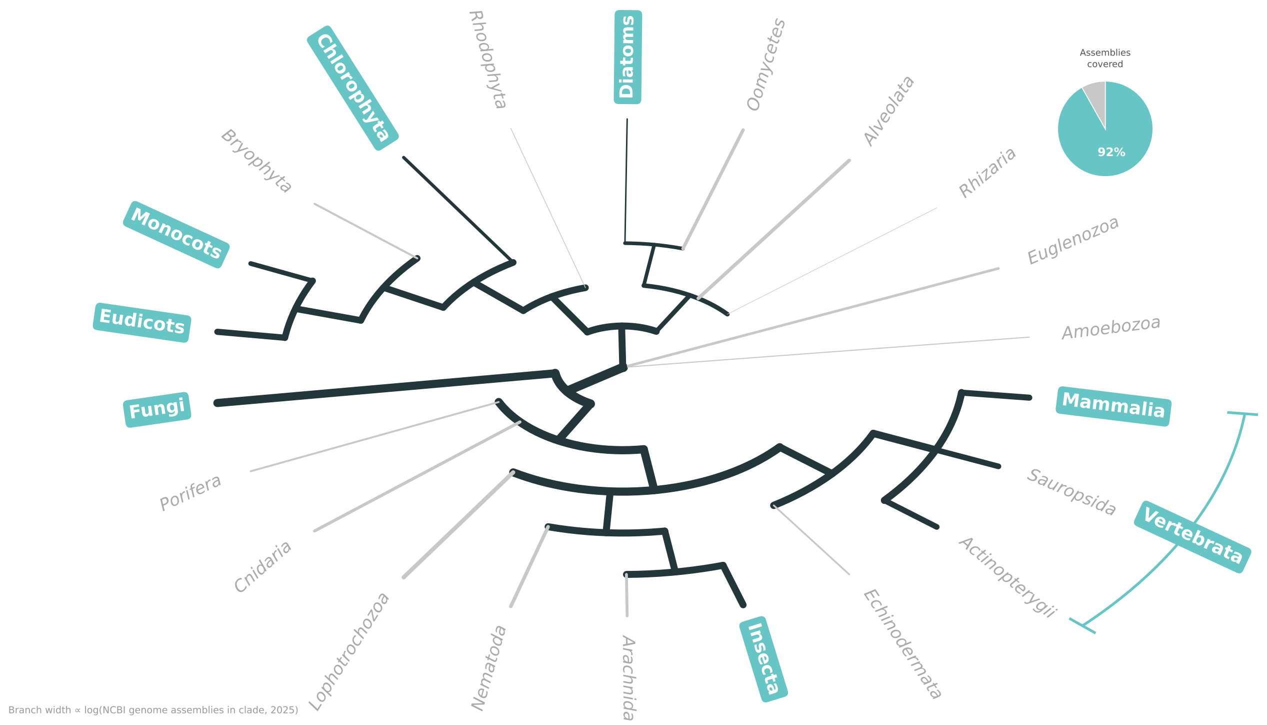

Figure 1: Visualization of taxonomic clade space covered by Tiberius. Data retrieved from NCBI on 2026-03-25.

Supplementary Methods

Tiberius Training

The Tiberius models were trained as described here: `https://github.com/Gaius-Augustus/Tiberius/blob/main/docs/training_large_data.md`, commit `46d7bc4`.

Accuracy metrics

Accuracy metrics were computed using `gffcompare` v0.12.10 on the basis of CDS features. Gffcompare was used so that instances from a prediction `prediction.gff` were only counted as true positives if they matched the reference `reference.gff` exactly. A tolerance of 3 bp was given to allow for differences in stop codon inclusion in the CDS features of the files.

```
gffcompare --strict-match -e 3 -T -r reference.gff prediction.gff
```

Benchmarking Tiberius

Tiberius v2.0.0 was used with one of the pretrained models for each test species, see Table 29. For a target genome `genome.fa` and a Tiberius model `${model}`, Tiberius was run with:

```
tiberius.py --genome genome.fa --model_cfg ${model}.yaml --out tiberius.gtf
```

Benchmarking ANNEVO

ANNEVO v2.2.1 was used with an appropriate pretrained model for each test species, see Table 29. For a target genome `genome.fa` and an ANNEVO model `${model}.pt`, ANNEVO was run with:

```
annotation.py --genome genome.fa --model_path ${model}.pt --threads 72 --output annevo.gff
```

Benchmarking Helixer

Helixer v0.3.4 was used with an appropriate pretrained model for each test species, see Table 29. For a target genome `genome.fa` and a Helixer model `${model}`, Helixer was run with:

```
Helixer.py --fasta-path genome.fa --lineage ${model} --gff-output-path helixer.gff
```

Benchmarking BRAKER3

BRAKER3 v3.0.8 was run using a softmasked genome, protein sequences and aligned RNA-Seq short reads as inputs. The proteomes of all training species from the Tiberius training of the clade of the target genome were used as the protein input `proteins.fa`.

The unmasked input genome `genome_dir/genome.fa` was softmasked for repeats using RED v2.0 with:

```
Red -gnm genome_dir/ -msk red/ -cor ${params.threads}
```

The short-read RNA-Seq input was generated with VARUS, which computes a `BAM` file with aligned reads that have sufficient coverage of the genome for genome annotation (resulting in `VARUS.bam`).

```
perl runVARUS.pl \
    --readFromTable=0 \
    --createindex=1 \
    --latinGenus=$genus_name \
    --latinSpecies=$species_name \
    --speciesGenome=genome.red.fa \
    --aligner=HISAT \
```

```
    --runThreadN 72 \
    --batchSize 50000 \
    --blockSize 5000 \
    --maxBatches 1000 \
    --mergeThreshold 10
```

BRAKER3 was then run with:

```
braker.pl \
    --genome=genome.red.fa \
    --prot_seq=proteins.fa \
    --threads=72 \
    --workingdir=braker3 \
    --bam VARUS.bam
```

Supplementary Tables

Species	Accession ID	Genome (Mb)	Annotation	#Genes	Species	Accession ID	Genome (Mb)	Annotation	#Genes
**Training**									
Cylcotella atomus	GCA_013187285.1	171	BRAKER	16,338	*Cyclotella choctawhatcheeana*	GCA_036939855.1	55	BRAKER	15,687
Conticribra guillardii	GCA_036939335.1	75	BRAKER	15,775	*Conticribra weissflogii*	GCA_036940025.1	130	BRAKER	14,524
Skeletonema menzelii	GCA_036940005.1	33	BRAKER	13,949	*Skeletonema potamos*	GCA_036940105.1	35	BRAKER	13,943
Discostella pseudostelligera	GCA_036940085.1	30	BRAKER	10,458	*Discostella stelligera*	GCA_036939735.1	59	BRAKER	13,154
Stephanodiscus triporus	GCA_036939755.1	58	BRAKER	13,345	*Cyclostephanos invisitatus*	GCA_036939675.1	58	BRAKER	12,825
Thalassiosira mediterranea	GCA_022925895.1	77	BRAKER	16,656	*Cylindrotheca closterium*	GCA_933822405.4	55.1	EMBL	23,336
Pseudo-nitschia multistriata	GCA_900660405.1	57	EMBL	11,733	*Mayamaea pseudoterrestris*	GCA_027923505.1	31	DDBJ	10,842
Seminavis robusta	GCA_903772945.1	126	EMBL	34,364	*Fragilaria crotonensis*	GCA_022925895.1	62	Genbank	25,842
Asterionella formosa	GCA_002256025.1	68	BRAKER	14,820	*Chaetoceros muelleri*	GCA_019693545.1	38	BRAKER	12,172
Chaetoceros tenuissimus	GCA_021927905.1	41	DDBJ	18,636					
**Validation**									
Nitzschia palea	GCA_019593585.1	41	BRAKER	15,386	*Skeletonema tropicum*	GCA_037178625.1	79	BRAKER	23,802
**Testing**									
Thalassiosira pseudonana	Filloramo *et al.*, 2021	34	AUGUSTUS/Geneious	14,830	*Phaeodactylum tricornutum*	GCF_000150955.2	27.6	ENSEMBL	11,909

Table 1: Accession ID, genome size, annotation source, and gene count for all species used for training and testing the Tiberius model for **Bacillariophyta**. The BRAKER annotations were provided by Nenasheva *et al.*, 2024, the genome and annotation of *Thalassiosira pseudonana* was downloaded from `https://doi.org/10.5683/SP2/ZDZQFE` (Filloramo *et al.*, 2021).

Species	Accession ID	Genome (Mb)	Annotation	#Genes	Species	Accession ID	Genome (Mb)	Annotation	#Genes
**Training**									
Ostreococcus lucimarinus CCE9901	GCF_000092065.1	13	RefSeq	6,381	*Chlamydomonas reinhardtii*	GCF_000002595.2	111.1	RefSeq	17,705
Volvox carteri f. nagariensis	GCF_000143455.1	138	RefSeq	12,362	*Picochlorum sp.* BPE2	GCA_025209345.1	14.9	Genbank	7,227
Astrephomene gubernaculifera	GCA_021605115.1	104	DDBJ	7,209	*Nannochloris sp. desiccata*	GCA_019044685.2	21.6	Genbank	9,191
Dunaliella primolecta	GCA_914767535.2	211	ensembl	10,240	*Chloropicon primus*	GCA_023205875.1	17.6	Genbank	8,513
Tetradesmus obliquus	GCA_030272155.1	105	Genbank	14,724					
**Validation**									
Micromonas commoda	GCF_000090985.2	21	RefSeq	9,201	*Gonium pectorale*	GCA_001584585.1	149	Genbank	16,290
**Testing**									
Edaphochlamys debaryana	GCA_016858145.1	142	Genbank	19,013	*Bathycoccus prasinos*	GCF_002220235.1	15	RefSeq	7,813

Table 2: Accession ID, genome size and gene count for all species used for training, validation, and testing the Tiberius model for **Chlorophyta**.

Species	Accession ID	Genome (Mb)	#Genes	Annotation source	Reference	DOI
**Training**						
Acorus americanus	586	374	26,508	Phytozome	Leebens-Mack et al. (2017)	doi:10.46936/10.25585/60001405
Agave tequilana	924	3,394	42,295	Phytozome	Xiaohan et al. (2017)	doi:10.46936/10.25585/60001139
Aquilegia coerulea	322	292	30,023	Phytozome	Filiault et al. (2018)	doi:10.7554/eLife.36426
Arundo donax	1047[1]	474	25,466	Phytozome	Blake et al. (2019)	doi:10.46936/10.25585/60001226
Beta vulgaris	782	535	21,587	Phytozome	McGrath et al. (2023)	doi:10.1093/dnares/dsac033
Brassica rapa	714	432	39,062	Phytozome	Greenham et al. (2018)	doi:10.46936/10.25585/60001202
Brocchinia micrantha	933[1]	338	20,842	Phytozome	Leebens-Mack et al. (2017)	doi:10.46936/10.25585/60001405
Capparis spinosa	851[1]	575	25,776	Phytozome	Harkess et al. (2020)	doi:10.46936/10.25585/60000482
Chasmanthium laxum	690	863	31,776	Phytozome	Schnable et al. (2013)	doi:10.46936/10.25585/60001029
Citrus limon	831[1]	321	25,726	Phytozome	-	-
Cleome violacea	585	279	21,850	Phytozome	Wing et al. (2011)	doi:10.46936/10.25585/60000980
Coffea arabica	871	1,025	51,583	Phytozome	Medrano et al. (2024)	doi:10.1093/g3journal/jkae262
Corylus avellana	858	350	32,431	Phytozome	Talbot et al. (2024)	doi:10.1093/g3journal/jkae021
Daucus carota	861	441	36,307	Phytozome	Coe et al. (2023)	doi:10.1038/s41477-023-01526-6
Dendrobium catenatum	GCA_002786265.1	1,061	22,642	NCBI	Zhang et al. (2017)	doi:10.1038/nature23897
Dioscorea alata	550	480	25,189	Phytozome	Bredeson et al. (2022)	doi:10.1038/s41467-022-29114-w
Eucalyptus grandis	891	571	35,929	Phytozome	Lötter et al. (2025)	doi:10.1093/g3journal/jkaf112
Fragaria vesca	677	219	34,006	Phytozome	Li et al. (2019)	doi:10.1038/s41438-019-0142-6
Glycine max	880	1,011	48,387	Phytozome	Espina et al. (2024)	doi:10.1111/tpj.17026
Gossypium hirsutum	695	2,296	75,366	Phytozome	-	-
Hydrocotyle leucocephala	768	756	32,364	Phytozome	Bertrand et al. (2019)	doi:10.46936/10.25585/60001311
Joinvillea ascendens	587	1,213	29,122	Phytozome	Leebens-Mack et al. (2017)	doi:10.46936/10.25585/60001405
Lactuca sativa	LsPKU06	2,593	45,981	Figshare	Wang et al. (2024)	doi:10.1016/j.xplc.2024.101011
Lindenbergia philippensis	689	426	26,721	Phytozome	Leebens-Mack et al. (2017)	doi:10.46936/10.25585/60001405
Malus domestica	WA 38 v1.0	652	54,235	Genome Database for Rosaceae	Zhang et al. (2024)	doi:10.1093/g3journal/jkae222
Manihot esculenta	671	637	32,805	Phytozome	Bredeson et al. (in prep.)	-
Mentha longifolia	-[1]	418	42,275	Oregon State University	Talbot et al. (in prep.)	-
Miscanthus sinensis	497	1,847	67,789	Phytozome	Mitros et al. (2020)	doi:10.1038/s41467-020-18923-6
Moringa oleifera	866[1]	271	20,761	Phytozome	Harkess et al. (2020)	doi:10.46936/10.25585/60000482
Musa acuminata	GCF_036884655.1	477	29,948	NCBI	Huang et al. (2023)	doi:10.1093/hr/uhad153
Myrothamnus flabellifolia	856	1,283	22,809	Phytozome	Marks et al. (2025)	doi:10.1111/nph.70700
Nicotiana tabacum	945	4,322	66,812	Phytozome	Wang et al. (2024)	doi:10.1016/j.molp.2024.01.008
Oryza sativa	499	378	35,594	Phytozome	Jain et al. (2019)	doi:10.1186/s12864-019-6262-4
Osyris compressa	823[1]	278	29,654	Phytozome	Leebens-Mack et al. (2017)	doi:10.46936/10.25585/60001405
Panicum virgatum	516	527	40,957	Phytozome	Lovell et al. (2021)	doi:10.1038/s41586-020-03127-1
Pharus latifolius	680	1,118	32,144	Phytozome	Leebens-Mack et al. (2017)	doi:10.46936/10.25585/60001405
Phoenix dactylifera	GCA_009389715.1	773	29,236	NCBI	Hazzouri et al. (2019)	doi:10.1038/s41467-019-12604-9
Setaria viridis	726	397	29,807	Phytozome	Mamidi et al. (2020)	doi:10.1038/s41587-020-0681-2
Sorghum bicolor	730	720	32,160	Phytozome	Morris et al. (2025)	doi:10.1101/2025.08.01.667986
Thinopyrum intermedium	770	4,303	41,523	Phytozome	Poland et al. (2015)	doi:10.46936/10.25585/60000844
Ulmus americana	881	1,461	25,621	Phytozome	-	-
Vitis vinifera	-[1]	488	40,588	Oregon State University	Talbot et al. (in prep.)	-
Yucca filamentosa	837	2,422	45,250	Phytozome	Heyduk et al. (2016)	doi:10.46936/10.25585/60001082
Zea mays	833	2,178	39,756	Phytozome	Hufford et al. (2021)	doi:10.1126/science.abg5289
Zingiber officinale	GCF_018446385.1	1,625	33,352	NCBI	-	-
**Validation**						
Populus trichocarpa	533	392	34,699	Phytozome	Tuskan et al. (2006)	doi:10.1126/science.1128691
Typha latifolia	853	215	22,107	Phytozome	Leebens-Mack et al. (2017)	doi:10.46936/10.25585/60001405
**Testing**						
Arabidopsis thaliana	447	119	27,655	Phytozome	Cheng et al. (2017)	doi:10.1111/tpj.13415
Brachypodium distachyon	GCF_000005505.3	271	25,534	NCBI	Vogel et al. (2010)	doi:10.1038/nature08747
Eschscholzia californica	828	384	29,637	Phytozome	Sierro et al. (2023)	doi:10.1093/plcell/koag039
Freycinetia multiflora	868	511	28,350	Phytozome	Leebens-Mack et al. (2017)	doi:10.46936/10.25585/60001405
Medicago truncatula	GCF_003473485.1	429	31,925	NCBI	Pecrix et al. (2018)	doi:10.1038/s41477-018-0286-7
Mimulus guttatus	797	339	25,113	Phytozome	Vallejo-Marín et al. (2025)	doi:10.1111/1755-0998.70012
Urochloa brizantha	897[1]	2,747	134,704	Phytozome	Ozias-Akins et al. (2020)	doi:10.46936/10.25585/60001353

Table 3: Genome assemblies and annotation sources used for training the **Mesangiospermae** model. [1]Not yet publicly released at the time of this study; used with permission from the data providers. All genomes and annotations used for training are available via Figshare (doi:10.6084/m9.figshare.32086728) for reproducibility purposes. Until their official public release on Phytozome or elsewhere, use of these data is subject to the Fort Lauderdale Accord and users should contact the original data providers before use in independent analyses.

Species	Accession ID	Genome (Mb)	#Genes	Species	Accession ID	Genome (Mb)	#Genes
**Training (1/2)**							
Aaosphaeria arxii	GCF_010015735.1	39	14,293	*Akanthomyces muscarius*	GCF_028009165.1	36	12,347
Alternaria atra	GCF_907166805.1	40	12,172	*Alternaria burnsii*	GCF_013036055.1	33	11,314
Alternaria rosae	GCF_020736505.1	34	12,727	*Amorphotheca resinae*	GCF_003019875.1	29	9,642
Annulohypoxylon maeteangense	GCF_022496945.1	38	11,144	*Annulohypoxylon truncatum*	GCF_022578515.1	38	11,304
Apiospora aurea	GCF_038362705.1	50	15,187	*Apiospora marii*	GCF_038362585.1	51	16,453
Apiotrichum porosum	GCF_003942205.1	25	9,719	*Aplosporella prunicola*	GCF_010093885.1	33	12,738
Ascochyta rabiei	GCF_004011695.2	41	11,642	*Aspergillus aculeatus*	GCF_001890905.1	35	11,152
Aspergillus alliaceus	GCF_009176365.1	40	13,336	*Aspergillus brunneoviolaceus*	GCF_003184695.1	37	12,377
Aspergillus caelatus	GCF_009193585.1	40	14,157	*Aspergillus campestris*	GCF_002847485.1	28	9,823
Aspergillus candidus	GCF_002847045.1	27	9,803	*Aspergillus chevalieri*	GCF_016861735.1	30	10,516
Aspergillus clavatus	GCF_000002715.2	28	9,375	*Aspergillus fijiensis*	GCF_003184825.1	37	12,324
Aspergillus fischeri	GCF_000149645.3	31	10,664	*Aspergillus glaucus*	GCF_001890805.1	28	11,428
Aspergillus heteromorphus	GCF_003184545.1	36	11,088	*Aspergillus homomorphus*	GCF_003184865.1	34	11,651
Aspergillus ibericus	GCF_003184845.1	33	11,983	*Aspergillus japonicus*	GCF_003184785.1	36	12,338
Aspergillus lentulus	GCF_010724455.1	30	10,506	*Aspergillus neoniger*	GCF_003184625.1	35	12,218
Aspergillus piperis	GCF_003184755.1	35	12,357	*Aspergillus puulaauensis*	GCF_016861865.1	34	13,748
Aspergillus saccharolyticus	GCF_003184585.1	31	10,375	*Aspergillus sclerotioniger*	GCF_003184525.1	37	12,603
Aspergillus steynii	GCF_002849105.1	38	13,146	*Aspergillus sydowii*	GCF_001890705.1	34	13,716
Aspergillus tubingensis	GCF_013340325.1	35	11,809	*Aspergillus udagawae*	GCF_001078395.1	32	11,009
Aspergillus uvarum	GCF_003184745.1	36	12,342	*Aspergillus vadensis*	GCF_003184925.1	36	12,402
Aspergillus versicolor	GCF_001890125.1	33	13,361	*Aspergillus viridinutans*	GCF_018404265.1	35	10,237
Aspergillus wentii	GCF_001890725.1	31	12,657	*Aureobasidium melanogenum*	GCF_000721775.1	26	10,582
Aureobasidium namibiae	GCF_000721765.1	25	10,259	*Aureobasidium pullulans*	GCF_000721785.1	30	11,844
Babjeviella inositovora	GCF_001661335.1	15	6,795	*Batrachochytrium dendrobatidis*	GCF_000203795.2	24	8,386
Beauveria bassiana	GCF_000280675.1	34	10,364	*Bipolaris oryzae*	GCF_000523455.1	31	12,000
Bipolaris victoriae	GCF_000527765.1	33	12,882	*Bipolaris zeicola*	GCF_000523435.1	31	12,848
Boeremia exigua	GCF_020726555.1	35	12,996	*Botrytis byssoidea*	GCF_014898295.1	43	12,204
Botrytis cinerea	GCF_000143535.2	43	11,698	*Botrytis deweyae*	GCF_014898535.1	44	12,465
Botrytis fragariae	GCF_013461495.1	42	12,345	*Botrytis porri*	GCF_014898465.1	47	12,076
Botrytis sinoallii	GCF_014898435.1	61	12,200	*Canariomyces notabilis*	GCF_033297395.1	31	11,102
Candida albicans	GCF_000182965.3	14	6,263	*Candida jiufengensis*	GCF_024610255.1	14	5,705
Candida orthopsilosis	GCF_000315875.1	13	5,782	*Candida parapsilosis*	GCF_000182765.1	13	5,950
Capronia coronata	GCF_000585585.1	26	9,231	*Capronia epimyces*	GCF_000585565.1	29	10,468
Cercospora kikuchii	GCF_019650295.1	34	13,001	*Chaetomium fimeti*	GCF_033439085.1	35	11,628
Chaetomium strumarium	GCF_033439665.1	32	10,401	*Chaetomium tenue*	GCF_020726465.1	34	11,734
Cladophialophora bantiana	GCF_000835475.1	37	12,817	*Cladophialophora carrionii*	GCF_000365165.1	29	10,428
Cladophialophora psammophila	GCF_000585535.1	39	13,421	*Cladophialophora yegresii*	GCF_000585515.1	28	10,118
Clavispora lusitaniae	GCF_014636115.1	12	5,702	*Coccidioides immitis*	GCF_000149335.2	29	9,905
Cokeromyces recurvatus	GCF_025118155.1	28	11,066	*Colletotrichum acutatum*	GCF_030867785.1	49	15,371
Colletotrichum godetiae	GCF_030913485.1	52	16,406	*Colletotrichum graminicola*	GCF_000149035.1	52	12,399
Colletotrichum karsti	GCF_011947395.1	52	13,328	*Colletotrichum navitas*	GCF_030913465.1	52	14,963
Colletotrichum orchidophilum	GCF_001831195.1	49	14,452	*Colletotrichum paranaense*	GCF_030867605.1	49	16,727
Colletotrichum phormii	GCF_030913505.1	52	15,598	*Colletotrichum tamarilloi*	GCF_030869305.1	52	17,330
Coniosporium apollinis	GCF_000281105.1	29	9,367	*Cryptococcus amylolentus*	GCF_001720205.1	20	8,503
Cryptococcus bacillisporus	GCF_000836335.1	18	6,809	*Cryptococcus decagattii*	GCF_036417295.1	18	6,671
Cryptococcus deuterogattii	GCF_002954075.1	18	6,673	*Cryptococcus gattii*	GCF_000185945.1	18	6,580
Cryptococcus neoformans	GCF_000091045.1	19	7,004	*Cryptococcus tetragattii*	GCF_000835755.1	18	6,807
Cryptococcus wingfieldii	GCF_001720155.1	20	8,314	*Cucurbitaria berberidis*	GCF_010015615.1	33	12,468
Cyberlindnera jadinii	GCF_001661405.1	13	6,184	*Cyphellophora europaea*	GCF_000365145.1	29	11,153
Cystobasidium minutum	GCF_039999085.1	21	7,862	*Daldinia caldariorum*	GCF_022478825.1	36	10,553
Daldinia decipiens	GCF_022478715.1	35	10,881	*Daldinia vernicosa*	GCF_022497035.1	36	10,827
Debaryomyces fabryi	GCF_001447935.2	12	6,025	*Debaryomyces hansenii*	GCF_000006445.2	12	6,520
Desarmillaria tabescens	GCF_030435595.1	75	19,381	*Diaporthe amygdali*	GCF_026229845.1	51	15,635
Dichomitus squalens	GCF_000275845.1	43	12,497	*Dichotomopilus funicola*	GCF_033296635.1	33	9,442
Didymella exigua	GCF_010094145.1	34	12,508	*Dioszegia hungarica*	GCF_025882075.1	21	8,321
Diplodia corticola	GCF_001883845.1	35	10,831	*Dipodascopsis tothii*	GCF_038497935.1	13	5,897
Dothidotthia symphoricarpi	GCF_010015815.1	34	11,821	*Drechmeria coniospora*	GCF_001625195.1	33	8,445
Drepanopeziza brunnea	GCF_022702325.1	52	9,615	*Durotheca rogersii*	GCF_024521655.1	48	10,623
Emericellopsis atlantica	GCF_019669845.1	27	10,066	*Eremothecium gossypii*	GCF_000091025.4	9	5,317
Eremothecium sinecaudum	GCF_001548555.1	9	4,970	*Exophiala aquamarina*	GCF_000709125.1	42	13,118
Exophiala dermatitidis	GCF_000230625.1	26	9,355	*Exophiala oligosperma*	GCF_000835515.1	38	11,938
Exophiala spinifera	GCF_000836115.1	33	12,110	*Exophiala viscosa*	GCF_022695815.1	28	11,369
Exophiala xenobiotica	GCF_000835505.1	31	12,077	*Fimicolochytrium jonesii*	GCF_025526975.1	31	10,236
Fomitiporia mediterranea	GCF_000271605.1	63	11,411	*Fonsecaea erecta*	GCF_001651985.1	35	12,120
Fonsecaea multimorphosa	GCF_000836435.1	33	12,405	*Fulvia fulva*	GCF_020509005.1	67	14,993
Fusarium fujikuroi	GCF_900079805.1	44	14,943	*Fusarium mangiferae*	GCF_900044065.1	46	16,140
Fusarium odoratissimum	GCF_000260195.1	47	16,975	*Fusarium proliferatum*	GCF_900067095.1	45	16,488
Fusarium pseudograminearum	GCF_000303195.2	37	12,445	*Fusarium redolens*	GCF_020744475.1	53	17,353
Fusarium subglutinans	GCF_013396075.1	44	14,039	*Fusarium vanettenii*	GCF_000151355.1	51	15,708
Fusarium venenatum	GCF_020744135.1	37	13,158	*Fusarium verticillioides*	GCF_000149555.1	42	16,290
Gaeumannomyces tritici	GCF_000145635.1	44	14,749	*Gamsiella multidivaricata*	GCF_025024155.1	38	12,419
Gilbertella persicaria	GCF_025201335.1	26	11,124	*Gloeophyllum trabeum*	GCF_000344685.1	37	11,883
Halteromyces radiatus	GCF_025201355.1	26	10,305	*Heterobasidion irregulare*	GCF_000320585.1	34	13,270
Hyphopichia burtonii	GCF_001661395.1	12	6,166	*Hypoxylon fragiforme*	GCF_022984875.1	36	10,382
Hypoxylon trugodes	GCF_022578975.1	39	12,128	*Ilyonectria robusta*	GCF_021365365.1	60	20,715

Table 4: Accession ID, genome size and gene count for all species used for training the Tiberius model for **Fungi** (1/2).

Species	Accession ID	Genome (Mb)	#Genes	Species	Accession ID	Genome (Mb)	#Genes
**Training (2/2)**							
Kazachstania africana	GCF_000304475.1	11	5,646	*Kickxella alabastrina*	GCF_025024165.1	22	7,463
Kluyveromyces lactis	GCF_000002515.2	11	5,335	*Kluyveromyces marxianus*	GCF_001417885.1	11	5,151
Kockiozyma suomiensis	GCF_038497685.1	14	6,203	*Kockovaella imperatae*	GCF_002102565.1	17	7,428
Komagataella phaffii	GCF_000027005.1	9	5,040	*Kuraishia capsulata*	GCF_000576695.1	11	5,988
Kwoniella bestiolae	GCF_000512585.2	25	9,214	*Kwoniella botswanensis*	GCF_036426115.1	23	8,705
Kwoniella dejecticola	GCF_000512565.2	24	8,719	*Kwoniella europaea*	GCF_036810445.1	23	8,670
Kwoniella mangrovensis	GCF_000507465.2	23	8,610	*Kwoniella newhampshirensis*	GCF_039105145.1	20	7,210
Kwoniella shandongensis	GCF_008629635.2	21	7,418	*Kwoniella shivajii*	GCF_035658355.1	22	7,975
Lachancea thermotolerans	GCF_000142805.1	10	5,384	*Laetiporus sulphureus*	GCF_001632365.1	40	13,840
Lasiodiplodia theobromae	GCF_012971845.1	44	13,054	*Lasiosphaeria miniovina*	GCF_030549165.1	49	15,242
Lentinula edodes	GCF_021015755.1	46	14,392	*Limtongia smithiae*	GCF_038497785.1	14	6,123
Linderina pennispora	GCF_002104995.1	26	9,583	*Lindgomyces ingoldianus*	GCF_010093535.1	69	16,000
Lipomyces chichibuensis	GCF_038497645.1	16	6,640	*Lipomyces doorenjongii*	GCF_038497915.1	19	7,414
Lipomyces japonicus	GCF_038497905.1	13	5,975	*Lipomyces oligophaga*	GCF_038497815.1	14	5,861
Lipomyces tetrasporus	GCF_029532465.1	21	8,094	*Lobosporangium transversale*	GCF_002105155.1	43	11,983
Macroventuria anomochaeta	GCF_010093625.1	33	13,082	*Metarhizium brunneum*	GCF_013426205.1	38	11,555
Metarhizium robertsii	GCF_000187425.2	42	11,688	*Mollisia scopiformis*	GCF_001500285.1	49	18,648
Morchella importuna	GCF_003444635.1	51	11,642	*Mucor mucedo*	GCF_025094135.1	46	14,226
Mucor velutinosus	GCF_035048745.1	40	13,535	*Mycotypha africana*	GCF_025528875.1	29	11,085
Mytilinidion resinicola	GCF_010093595.1	47	16,878	*Myxozyma melibiosi*	GCF_037950935.1	15	6,393
Nakaseomyces glabratus	GCF_010111755.1	13	5,508	*Nannizzia gypsea*	GCF_000150975.2	23	9,029
Naumovozyma dairenensis	GCF_000227115.2	14	5,814	*Neoarthrinium moseri*	GCF_022829205.1	44	13,929
Neohortaea acidophila	GCF_010093505.1	20	9,858	*Neurospora crassa*	GCF_000182925.2	41	10,590
Neurospora tetraspora	GCF_033439725.1	44	11,717	*Paecilomyces variotii*	GCF_004022145.1	30	9,415
Paraphaeosphaeria sporulosa	GCF_001642045.1	38	14,839	*Parathielavia appendiculata*	GCF_033297405.1	33	12,100
Penicillium alfredii	GCF_028826965.1	27	10,160	*Penicillium angulare*	GCF_028827245.1	38	13,393
Penicillium argentinense	GCF_028826775.1	34	12,141	*Penicillium atrosanguineum*	GCF_028827265.1	29	10,995
Penicillium bovifimosum	GCF_028826915.1	27	10,392	*Penicillium brevicompactum*	GCF_028827555.1	35	12,371
Penicillium canariense	GCF_028826845.1	32	10,797	*Penicillium cataractarum*	GCF_028827025.1	37	12,826
Penicillium chrysogenum	GCF_028827035.1	32	11,965	*Penicillium cinerascens*	GCF_028974065.1	29	11,023
Penicillium concentricum	GCF_028827145.1	30	11,702	*Penicillium coprophilum*	GCF_028826855.1	29	10,975
Penicillium daleae	GCF_028827525.1	40	12,820	*Penicillium digitatum*	GCF_016767815.1	26	9,238
Penicillium expansum	GCF_000769745.1	32	11,058	*Penicillium griseofulvum*	GCF_001561935.1	29	9,628
Penicillium hispanicum	GCF_028827665.1	28	10,111	*Penicillium macrosclerotiorum*	GCF_028827735.1	34	11,709
Penicillium malachiteum	GCF_028827825.1	36	13,213	*Penicillium nucicola*	GCF_028828085.1	31	11,520
Penicillium odoratum	GCF_028828045.1	32	12,000	*Penicillium oxalicum*	GCF_001723175.1	31	9,966
Penicillium paradoxum	GCF_028828445.1	29	9,841	*Penicillium psychrosexuale*	GCF_028828465.1	28	10,478
Penicillium pulvis	GCF_028828015.1	33	12,164	*Penicillium robsamsonii*	GCF_028829455.1	29	11,257
Penicillium solitum	GCF_028829755.1	33	12,723	*Penicillium soppii*	GCF_028829465.1	32	12,167
Penicillium subrubescens	GCF_028828155.1	40	13,459	*Penicillium taxi*	GCF_028828555.1	28	10,052
Penicillium verhagenii	GCF_028828195.1	32	11,667	*Phycomyces blakesleeanus*	GCF_001638985.1	54	16,850
Phyllosticta capitalensis	GCF_038381095.1	32	12,229	*Phyllosticta citribraziliensis*	GCF_038025025.1	31	10,847
Plenodomus lingam	GCF_022343315.1	42	11,989	*Pleurotus ostreatus*	GCF_014466165.1	35	11,849
Polychytrium aggregatum	GCF_025602895.1	65	11,051	*Postia placenta*	GCF_002117355.1	42	12,716
Priceomyces carsonii	GCF_900007245.1	12	6,179	*Pseudocercospora fijiensis*	GCF_000340215.1	74	13,125
Pseudogymnoascus verrucosus	GCF_001662655.1	30	10,799	*Pseudomassariella vexata*	GCF_002105095.1	45	12,950
Pseudozyma flocculosa	GCF_000417875.1	23	6,877	*Pseudozyma hubeiensis*	GCF_000403515.1	18	7,619
Psilocybe cubensis	GCF_017499595.1	46	13,556	*Purpureocillium lilacinum*	GCF_023168085.1	39	10,642
Purpureocillium takamizusanense	GCF_022605165.1	36	10,859	*Pyricularia grisea*	GCF_004355905.1	45	12,441
Pyricularia oryzae	GCF_000002495.2	41	13,184	*Pyricularia pennisetigena*	GCF_004337985.1	49	11,428
Radiomyces spectabilis	GCF_025331425.1	30	11,044	*Ramularia collo-cygni*	GCF_900074925.1	32	11,544
Rhizophagus irregularis	GCF_026210795.1	147	30,147	*Rhizopus microsporus*	GCF_002708625.1	26	10,958
Rhodotorula toruloides	GCF_000320785.1	20	8,171	*Saccharomyces cerevisiae*	GCF_000146045.2	12	6,477
Saccharomyces kudriavzevii	GCF_947243775.1	12	5,880	*Saccharomyces mikatae*	GCF_947241705.1	12	5,849
Saccharomyces paradoxus	GCF_002079055.1	12	5,840	*Saitoella complicata*	GCF_001661265.1	14	7,200
Saprochaete ingens	GCF_902498895.1	21	6,475	*Scheffersomyces amazonensis*	GCF_036850825.1	14	6,209
Scheffersomyces stipitis	GCF_000209165.1	15	5,819	*Scheffersomyces xylosifermentans*	GCF_036884685.1	17	6,350
Schizosaccharomyces cryophilus	GCF_000004155.1	12	5,494	*Schizosaccharomyces japonicus*	GCF_000149845.2	12	5,224
Schizosaccharomyces octosporus	GCF_000150505.1	12	5,347	*Schizosaccharomyces pombe*	GCF_000002945.2	13	12,645
Sodiomyces alkalinus	GCF_003711515.1	43	9,625	*Sordaria macrospora*	GCF_033870435.1	39	10,804
Sparassis crispa	GCF_003851025.1	39	13,339	*Sphaerulina musiva*	GCF_000320565.1	29	10,228
Spizellomyces punctatus	GCF_000182565.1	24	9,169	*Stereum hirsutum*	GCF_000264905.1	47	14,449
Suhomyces tanzawaensis	GCF_001661415.1	13	6,143	*Suillus bovinus*	GCF_016758785.1	47	13,655
Suillus clintonianus	GCF_016758775.1	47	15,647	*Suillus fuscotomentosus*	GCF_016647785.1	80	18,660
Suillus paluster	GCF_016628075.1	62	16,708	*Suillus plorans*	GCF_016647745.1	52	16,525
Suillus subalutaceus	GCF_016647625.1	65	17,233	*Synchytrium microbalum*	GCF_006535985.1	26	6,304
Talaromyces proteolyticus	GCF_021365285.1	38	13,665	*Talaromyces stipitatus*	GCF_000003125.1	36	12,572
Torulaspora delbrueckii	GCF_000243375.1	9	5,174	*Torulaspora globosa*	GCF_014133895.1	9	5,129
Trametes versicolor	GCF_000271585.1	45	14,562	*Trematosphaeria pertusa*	GCF_010094035.1	48	17,364
Trichoderma aggressivum	GCF_033847375.1	39	10,998	*Trichoderma breve*	GCF_028502605.1	39	11,590
Trichoderma harzianum	GCF_003025095.1	41	14,294	*Trichoderma reesei*	GCF_000167675.1	33	9,116
Trichoderma virens	GCF_000170995.1	39	12,382	*Truncatella angustata*	GCF_020726525.1	47	14,971
Umbelopsis ramanniana	GCF_025399195.1	23	10,039	*Ustilaginoidea virens*	GCF_000687475.1	37	8,297
Ustilago hordei	GCF_900519145.1	27	7,703	*Ustilago maydis*	GCF_000328475.2	20	6,907
Venustampulla echinocandica	GCF_003357145.1	34	10,707	*Westerdykella ornata*	GCF_010094085.1	27	10,501
Wickerhamomyces anomalus	GCF_001661255.1	14	6,571	*Xylaria bambusicola*	GCF_022495145.1	46	12,324
Yarrowia lipolytica	GCF_014490615.1	21	7,247	*Zasmidium cellare*	GCF_010093935.1	38	16,072
Zychaea mexicana	GCF_025766255.1	51	14,134	*Zygotorulaspora mrakii*	GCF_013402915.1	10	5,246

Table 5: Accession ID, genome size and gene count for all species used for training and testing the Tiberius model for **Fungi** (2/2).

Species	Accession ID	Genome (Mb)	#Genes	Species	Accession ID	Genome (Mb)	#Genes
**Validation**							
Alternaria alternata	GCF_001642055.1	33	13,577	*Alternaria arborescens*	GCF_004154835.1	34	12,895
Aspergillus aculeatinus	GCF_003184765.1	36	12,350	*Aspergillus costaricensis*	GCF_003184835.1	37	12,234
Aspergillus eucalypticola	GCF_003184535.1	35	12,127	*Aspergillus pseudonomiae*	GCF_009193645.1	38	13,621
Aspergillus pseudoviridinutans	GCF_018340605.1	33	11,496	*Aspergillus ruber*	GCF_000600275.1	26	10,229
Aureobasidium subglaciale	GCF_000721755.1	26	10,792	*Bipolaris sorokiniana*	GCF_000338995.1	34	12,304
Candida pseudojiufengensis	GCF_024610245.1	14	5,846	*Cercospora beticola*	GCF_033473495.1	36	13,212
Colletotrichum abscissum	GCF_030869225.1	54	17,223	*Colletotrichum chrysophilum*	GCF_026319265.1	56	14,530
Colletotrichum costaricense	GCF_030867565.1	52	17,065	*Colletotrichum destructivum*	GCF_034447905.1	52	15,627
Colletotrichum fructicola	GCF_000319635.2	60	17,388	*Coprinopsis cinerea*	GCF_000182895.1	36	13,657
Cutaneotrichosporon cavernicola	GCF_030864355.1	20	7,753	*Daldinia loculata*	GCF_022478755.1	38	10,988
Diaporthe citri	GCF_014595645.1	64	15,918	*Epithele typhae*	GCF_022376455.1	56	13,990
Fonsecaea pedrosoi	GCF_000835455.1	35	12,573	*Fusarium flagelliforme*	GCF_020744385.1	40	13,910
Fusarium musae	GCF_019915245.1	44	13,962	*Fusarium poae*	GCF_019609905.1	44	14,125
Geosmithia morbida	GCF_012550715.1	27	7,826	*Glarea lozoyensis*	GCF_000409485.1	40	13,081
Guyanagaster necrorhizus	GCF_019112545.1	54	14,487	*Kalmanozyma brasiliensis*	GCF_000497045.1	17	6,542
Kwoniella dendrophila	GCF_036810415.1	23	8,131	*Kwoniella pini*	GCF_000512605.1	21	7,931
Lichtheimia ornata	GCF_029851405.1	38	13,169	*Lipomyces arxii*	GCF_038497795.1	12	5,792
Marasmius oreades	GCF_018924745.1	44	13,890	*Microdochium trichocladiopsis*	GCF_020744255.1	49	16,222
Moesziomyces antarcticus	GCF_000747765.1	18	6,885	*Morchella sextelata*	GCF_020137385.1	54	13,182
Naumovozyma castellii	GCF_000237345.1	11	5,867	*Neurospora hispaniola*	GCF_033458365.1	41	10,876
Paracoccidioides lutzii	GCF_000150705.2	33	8,953	*Penicilliopsis zonata*	GCF_001890105.1	26	10,027
Penicillium antarcticum	GCF_028974205.1	31	11,217	*Penicillium crustosum*	GCF_028827405.1	33	12,327
Penicillium diatomitis	GCF_028827545.1	34	9,582	*Penicillium hordei*	GCF_028827395.1	34	12,337
Penicillium longicatenatum	GCF_028827895.1	32	11,974	*Penicillium rubens*	GCF_028828025.1	30	11,613
Penicillium verrucosum	GCF_028828655.1	33	11,554	*Penicillium vulpinum*	GCF_028829585.1	32	11,510
Pisolithus orientalis	GCF_024613125.1	58	12,436	*Rhinocladiella mackenziei*	GCF_000835555.1	32	11,418
Rhodofomes roseus	GCF_022264815.1	38	13,143	*Schizophyllum commune*	GCF_000143185.2	39	16,467
Schizosaccharomyces osmophilus	GCF_027921745.1	11	5,574	*Suillus discolor*	GCF_016758755.1	61	16,641
Suillus subaureus	GCF_016647635.1	58	15,900	*Tetrapisispora phaffii*	GCF_000236905.1	12	5,462
Wickerhamomyces ciferrii	GCF_000313485.1	16	6,702	*Zygosaccharomyces rouxii*	GCF_000026365.1	10	5,323
**Testing**							
Agaricus bisporus	GCF_000300555.1	33	11,443	*Cryphonectria parasitica*	GCF_011745365.1	44	11,709
Parastagonospora nodorum	GCF_000146915.1	37	16,114	*Puccinia striiformis*	GCF_021901695.1	89	17,813
Punctularia strigosozonata	GCF_000264995.1	34	11,644	*Tilletiopsis washingtonensis*	GCF_003144115.1	19	7,053

Table 6: Accession ID, genome size and gene count for all species used for validating and testing the Tiberius model for **Fungi**.

Species	Accession ID	Genome (Mb)	Annotation	#Genes	Species	Accession ID	Genome (Mb)	Annotation	#Genes
**Training**									
Aedes aegypti	GCF_002204515.2	1,279	RefSeq	14,731	*Agrotis ipsilon*	GCA_028554685.2	515	BRAKER	11,962
Amphipyra tragopoginis	GCA_905220435.1	806	BRAKER	18,183	*Anabrus simplex*	GCF_040414725.1	6,424	RefSeq	14,968
Anastrepha ludens	GCF_028408465.1	821	BRAKER	18,103	*Andrena minutula*	GCA_929113495.1	380	BRAKER	12,657
Anopheles arabiensis	GCF_016920715.1	257	BRAKER	12,969	*Anopheles farauti*	GCA_000473445.2	183	BRAKER	11,884
Anopheles stephensi	GCF_013141755.1	243	BRAKER	12,990	*Aphis glycines*	GCA_009761285.1	308	BRAKER	16,004
Apis mellifera	GCF_003254395.2	225	BRAKER	11,040	*Aporophyla lueneburgensis*	GCA_932294355.1	978	BRAKER	23,312
Arctia plantaginis	GCA_902825455.1	578	BRAKER	16,231	*Armigeres subalbatus*	GCF_024139115.2	1,336	RefSeq	19,126
Bactrocera dorsalis	GCF_023373825.1	530	BRAKER	18,592	*Bactrocera minax*	GCA_021498325.1	324	BRAKER	12,187
Bactrocera oleae	GCF_001188975.3	485	BRAKER	14,673	*Bactrocera tryoni*	GCF_016617805.1	571	BRAKER	17,801
Bemisia tabaci	GCF_001854935.1	615	BRAKER	14,377	*Bicyclus anynana*	GCF_947172395.1	457	RefSeq	13,653
Bombus impatiens	GCF_000188095.3	247	BRAKER	12,389	*Bradysia coprophila*	GCA_014529535.2	310	BRAKER	19,444
Calliphora vicina	GCA_958450345.1	707	BRAKER	16,315	*Camponotus floridanus*	GCF_003227725.1	284	BRAKER	13,038
Cardiocondyla obscurior	GCA_019399895.1	193	BRAKER	15,059	*Ceratitis capitata*	GCF_000347755.3	436	BRAKER	13,455
Chilo suppressalis	GCA_902850365.2	783	BRAKER	22,590	*Chrysoperla carnea*	GCF_905475395.1	560	BRAKER	14,441
Cimex lectularius	GCF_000648675.2	511	BRAKER	13,021	*Cochliomyia hominivorax*	GCA_004302925.2	534	BRAKER	12,788
Cryptotermes secundus	GCF_002891405.2	1,019	BRAKER	14,614	*Culex quinquefasciatus*	GCF_015732765.1	573	BRAKER	15,246
Dendroctonus ponderosae	GCF_020466585.1	224	BRAKER	14,204	*Dermacentor andersoni*	GCF_023375885.2	2,425	RefSeq	20,700
Diuraphis noxia	GCF_001186385.1	395	BRAKER	23,559	*Drosophila birchii*	GCA_008042755.1	157	BRAKER	16,937
Drosophila grimshawi	GCF_018153295.1	191	BRAKER	12,744	*Drosophila innubila*	GCA_004354385.2	166	BRAKER	15,008
Drosophila navojoa	GCF_001654015.2	147	BRAKER	12,770	*Drosophila obscura*	GCF_018151105.1	180	BRAKER	16,091
Drosophila suzukii	GCF_013340165.1	268	BRAKER	17,364	*Ennomos fuscantarius*	GCA_905220475.3	445	BRAKER	14,282
Episyrphus balteatus	GCF_945859705.1	535	BRAKER	14,004	*Formica exsecta*	GCF_003651465.1	278	BRAKER	18,914
Frankliniella occidentalis	GCF_000697945.3	406	BRAKER	22,507	*Galleria mellonella*	GCF_026898425.1	472	BRAKER	13,576
Glossina palpalis	GCA_000818775.1	380	BRAKER	9,740	*Gryllus bimaculatus*	GCA_017312745.1	1,658	BRAKER	11,991
Halyomorpha halys	GCA_000696795.3	1,150	BRAKER	16,536	*Harpegnathos saltator*	GCF_003227715.2	334	BRAKER	14,955
Helicoverpa zea	GCF_022581195.2	375	BRAKER	16,321	*Hermetia illucens*	GCF_905115235.1	1,005	BRAKER	15,393
Hormaphis cornu	GCA_017140985.1	320	BRAKER	14,080	*Hycleus cichorii*	GCA_013841215.1	99	BRAKER	8,417
Ips nitidus	GCA_018691245.2	231	BRAKER	21,165	*Laodelphax striatellus*	GCA_017141395.1	510	BRAKER	13,701
Lasiommata megera	GCA_928268935.1	488	BRAKER	12,772	*Leptopilina boulardi*	GCF_019393585.1	355	BRAKER	14,452
Lymantria dispar	GCA_016802235.1	998	BRAKER	19,022	*Malaya genurostris*	GCF_030247185.1	831	RefSeq	13,645
Megalopta genalis	GCF_011865705.1	390	BRAKER	10,806	*Melanaphis sacchari*	GCF_002803265.2	300	BRAKER	13,106
Melitaea cinxia	GCF_905220565.1	499	BRAKER	19,514	*Metopolophium dirhodum*	GCA_019925205.1	481	BRAKER	22,550
Monomorium pharaonis	GCF_013373865.1	326	BRAKER	17,086	*Musca domestica*	GCF_000371365.1	750	BRAKER	13,580
Mythimna separata	GCA_029852925.1	688	BRAKER	17,248	*Nasonia vitripennis*	GCF_009193385.2	297	BRAKER	21,342
Nezara viridula	GCA_928085145.1	1,185	BRAKER	19,624	*Nicrophorus vespilloides*	GCF_001412225.1	195	BRAKER	13,883
Onthophagus taurus	GCF_000648695.1	267	BRAKER	19,160	*Ooceraea biroi*	GCF_003672135.1	224	BRAKER	14,589
Operophtera brumata	GCA_932527175.1	619	BRAKER	15,652	*Oryctes rhinoceros*	GCA_020654165.1	377	BRAKER	11,931
Ostrinia furnacalis	GCF_004193835.2	435	BRAKER	15,189	*Pediculus humanus*	GCF_000006295.1	111	BRAKER	8,810
Periplaneta americana	GCA_025594305.2	3,056	BRAKER	19,261	*Phlebotomus papatasi*	GCF_024763615.1	352	BRAKER	10,881
Plodia interpunctella	GCF_027563975.1	291	BRAKER	14,816	*Pogonomyrmex californicus*	GCA_024349325.1	252	BRAKER	28,178
Polypedilum vanderplanki	GCA_018290095.1	119	BRAKER	14,780	*Propsilocerus akamusi*	GCA_018397935.1	86	BRAKER	12,517
Rhogogaster chlorosoma	GCA_944452935.1	255	BRAKER	21,737	*Rhopalosiphum padi*	GCA_020882245.1	338	BRAKER	13,277
Rhynchophorus ferrugineus	GCA_030347505.1	779	BRAKER	12,216	*Scaptomyza flava*	GCA_030179655.1	330	BRAKER	14,660
Schizaphis graminum	GCA_020882235.1	499	BRAKER	28,035	*Schlechtendalia chinensis*	GCA_019022885.1	280	BRAKER	17,424
Sipha flava	GCF_003268045.1	353	BRAKER	9,105	*Sitobion avenae*	GCA_019425605.1	393	BRAKER	10,662
Sitophilus oryzae	GCF_002938485.1	771	BRAKER	20,600	*Sogatella furcifera*	GCA_017141385.1	564	BRAKER	11,007
Solenopsis invicta	GCF_016802725.1	378	BRAKER	18,058	*Spodoptera litura*	GCF_002706865.2	430	BRAKER	16,178
Stomoxys calcitrans	GCF_963082655.1	1,071	RefSeq	15,234	*Temnothorax longispinosus*	GCA_030848805.1	298	BRAKER	20,311
Thrips palmi	GCF_012932325.1	238	BRAKER	14,785	*Thymelicus sylvestris*	GCA_911387775.1	471	BRAKER	15,050
Trichoplusia ni	GCF_003590095.1	368	BRAKER	15,313	*Zeugodacus cucurbitae*	GCF_028554725.1	439	BRAKER	15,987
**Test**									
Bombyx mori	GCF_030269925.1	462	RefSeq	18,210	*Cataglyphis hispanica*	GCF_021464435.1	206	RefSeq	11,502
Colias croceus	GCF_905220415.1	325	RefSeq	15,772	*Danaus plexippus*	GCF_018135715.1	245	RefSeq	14,790
Drosophila melanogaster	GCF_000001215.4	144	RefSeq	17,872	*Leptidea sinapis*	GCF_905404315.1	686	RefSeq	15,767
Nymphalis io	GCF_905147045.1	384	RefSeq	13,823	*Osmia bicornis*	GCF_907164935.1	223	RefSeq	12,706
Tribolium castaneum	GCF_031307605.1	242	RefSeq	15,518	*Vanessa cardui*	GCF_905220365.1	425	RefSeq	15,000
Zerene cesonia	GCF_012273895.1	266	RefSeq	12,772					
**Validation**									
Anopheles marshallii	GCF_943734725.1	226	RefSeq	13,358	*Danaus plexippus*	GCF_018135715.1	245	BRAKER	14,790
Drosophila ananassae	GCF_017639315.1	214	BRAKER	16,982	*Drosophila virilis*	GCF_030788295.1	189	BRAKER	15,650
Tenebrio molitor	GCF_963966145.1	277	BRAKER	19,578					

Table 7: Accession ID, genome size and gene count for all species used for training, validating and testing the Tiberius model for **Insecta**. Annotation source refers to the annotation used during training (BRAKER = AUGUSTUS/GeneMark pipeline; RefSeq = NCBI Gnomon pipeline).

Species	Accession ID	Genome (Mb)	#Genes	Species	Accession ID	Genome (Mb)	#Genes
**Training**							
Acipenser ruthenus	GCF_902713425.1	1,900	80,041	*Alligator mississippiensis*	GCF_030867095.1	2,347	31,016
Alosa sapidissima	GCF_018492685.1	904	47,628	*Amblyraja radiata*	GCF_010909765.2	2,559	25,978
Anguilla anguilla	GCF_013347855.1	979	31,451	*Anolis sagrei*	GCF_037176765.1	1,951	23,363
Apus apus	GCF_020740795.1	1,100	16,724	*Bombina bombina*	GCF_027579735.1	10,020	31,613
Carcharodon carcharias	GCF_017639515.1	4,286	25,164	*Corvus hawaiiensis*	GCF_020740725.1	1,152	21,647
Dendropsophus ebraccatus	GCF_027789765.1	2,215	37,424	*Denticeps clupeoides*	GCF_900700375.1	567	40,127
Elgaria multicarinata webbii	GCF_023053635.1	1,791	21,748	*Emys orbicularis*	GCF_028017835.1	2,310	22,788
Erpetoichthys calabaricus	GCF_900747795.2	3,614	27,285	*Esox lucius*	GCF_011004845.1	919	30,105
Eublepharis macularius	GCF_028583425.1	2,237	24,025	*Falco peregrinus*	GCF_023634155.1	1,312	19,918
Gavia stellata	GCF_030936135.1	1,321	17,980	*Labrus mixtus*	GCF_963584025.1	741	41,941
Latimeria chalumnae	GCF_037176945.1	2,948	28,938	*Lepisosteus oculatus*	GCF_040954835.1	1,201	49,180
Megalops cyprinoides	GCF_013368585.1	959	27,986	*Mobula birostris*	GCF_030028105.1	4,015	27,860
Narcine bancroftii	GCF_036971445.1	3,328	30,148	*Osmerus eperlanus*	GCF_963692335.1	509	34,446
Parambassis ranga	GCF_900634625.1	551	27,951	*Pelobates fuscus*	GCF_036172605.1	3,606	49,915
Podarcis raffonei	GCF_027172205.1	1,513	26,204	*Rana temporaria*	GCF_905171775.1	4,111	38,300
Rhinatrema bivittatum	GCF_901001135.1	5,319	28,114	*Scleropages formosus*	GCF_900964775.1	785	27,406
Solea solea	GCF_958295425.1	644	37,145	*Sparus aurata*	GCF_900880675.1	834	32,006
Aotus nancymaae	GCF_000952055.2	2,862	31,334	*Camelus bactrianus*	GCF_000767855.1	1,993	29,764
Canis lupus familiaris	GCF_000002285.3	2,411	36,943	*Cavia porcellus*	GCF_000151735.1	2,723	31,557
Cebus capucinus	GCF_001604975.1	2,718	36,525	*Ceratotherium simum*	GCF_000283155.1	2,464	25,301
Chinchilla lanigera	GCF_000276665.1	2,391	30,122	*Condylura cristata*	GCF_000260355.1	1,770	19,773
Desmodus rotundus	GCF_002940915.1	2,064	29,514	*Dipodomys ordii*	GCF_000151885.1	2,236	23,237
Enhydra lutris	GCF_002288905.1	2,455	24,129	*Eptesicus fuscus*	GCF_000308155.1	2,027	30,638
Equus caballus	GCF_000002305.2	2,475	26,780	*Heterocephalus glaber*	GCF_000247695.1	2,618	37,544
Ictidomys tridecemlineatus	GCF_000236235.1	2,478	26,378	*Jaculus jaculus*	GCF_000280705.1	2,835	25,112
Loxodonta africana	GCF_000001905.1	3,197	28,964	*Marmota marmota*	GCF_001458135.1	2,511	27,672
Microcebus murinus	GCF_000165445.2	2,487	31,964	*Microtus ochrogaster*	GCF_000317375.1	2,287	26,397
Mus musculus	GCF_000001635.26	2,731	49,997	*Mus pahari*	GCF_900095145.1	2,475	29,483
Neomonachus schauinslandi	GCF_002201575.1	2,401	24,767	*Ochotona princeps*	GCF_000292845.1	2,230	21,216
Octodon degus	GCF_000260255.1	2,996	39,965	*Odobenus rosmarus*	GCF_000321225.1	2,400	24,996
Otolemur garnettii	GCF_000181295.1	2,520	31,917	*Panthera pardus*	GCF_001857705.1	2,578	34,200
Propithecus coquereli	GCF_000956105.1	2,798	22,796	*Puma concolor*	GCF_003327715.1	2,433	23,982
Rattus norvegicus	GCF_000001895.5	2,870	39,469	*Saimiri boliviensis*	GCF_000235385.1	2,609	27,148
Sorex araneus	GCF_000181275.1	2,423	21,793	*Trichechus manatus*	GCF_000243295.1	3,104	25,210
**Testing**							
Archocentrus centrarchus	GCF_007364275.1	933	29,275	*Betta splendens*	GCF_900634795.4	427	28,938
Gallus gallus	GCF_016700215.2	1,051	25,564	*Pristiophorus japonicus*	GCF_044704955.1	5,923	55,857
Takifugu rubripes	GCF_901000725.2	384	27,434	*Zootoca vivipara*	GCF_963506605.1	1,440	22,823

Table 8: Accession ID, genome size and gene count for all species according to the corresponding NCBI RefSeq annotation used for training and testing the Tiberius model for **Vertebrata**.

Species	Accession ID	Genome (Mb)	#Genes
**Testing**			
Bos taurus	GCF_002263795.3	2,771	37,063
Delphinapterus leucas	GCF_002288925.2	2,363	27,561
Homo sapiens	GCF_000001405.40	3,099	59,792

Table 9: Accession ID, genome size and gene count for all species used for testing the Tiberius model for **Mammalia**. The Mammalia training and validation is described in the original Tiberius paper (Gabriel et al., 2024) and remained unchanged.

Table 10: Runtime benchmark for Chlorophyta.

Species	Tool	Runtime (h:mm)
Bathycoccus prasinos	Helixer	0:04
	Tiberius	0:01
	BRAKER3	10:34
Edaphochlamys debaryana	Helixer	0:25
	Tiberius	0:04
	BRAKER3	24:24

Table 11: Runtime benchmark for Bacillariophyta.

Species	Tool	Runtime (h:mm)
Phaeodactylum tricornutum	Tiberius	0:01
	BRAKER3	19:57
Thalassiosira pseudonana	Tiberius	0:01
	BRAKER3	18:30

Table 12: Runtime benchmark for Mesangiospermae.

Species	Tool	Runtime (h:mm)
Arabidopsis thaliana	Helixer	0:15
	ANNEVO	0:05
	Tiberius	0:04
	BRAKER3	33:02
Brachypodium stacei	Helixer	0:38
	ANNEVO	0:10
	Tiberius	0:07
	BRAKER3	4:23
Eschscholzia californica	Helixer	0:53
	ANNEVO	0:12
	Tiberius	0:10
	BRAKER3	31:29
Medicago truncatula	Helixer	0:59
	ANNEVO	0:14
	Tiberius	0:11
	BRAKER3	5:24
Freycinetia multiflora	Helixer	1:23
	ANNEVO	0:12
	Tiberius	0:13
	BRAKER3	6:02
Mimulus guttatus	Helixer	0:41
	ANNEVO	0:09
	Tiberius	0:08
	BRAKER3	46:36
Urochloa brizantha	Helixer	6:16
	ANNEVO	1:34
	Tiberius	1:04
	BRAKER3	16:06

Table 13: Runtime benchmark for Insecta.

Species	Tool	Runtime (h:mm)
Bombyx mori	Helixer	1:39
	ANNEVO	0:15
	Tiberius	0:16
	BRAKER3	63:28
Cataglyphis hispanica	Helixer	0:52
	ANNEVO	0:07
	Tiberius	0:08
	BRAKER3	36:48
Colias croceus	Helixer	1:12
	ANNEVO	0:10
	Tiberius	0:11
	BRAKER3	51:57
Danaus plexippus	Helixer	0:49
	ANNEVO	0:09
	Tiberius	0:09
	BRAKER3	46:13
Drosophila melanogaster	Helixer	0:54
	ANNEVO	0:07
	Tiberius	0:09
	BRAKER3	34:32
Leptidea sinapis	Helixer	2:31
	ANNEVO	0:21
	Tiberius	0:22
	BRAKER3	70:55
Nymphalis io	Helixer	1:20
	ANNEVO	0:13
	Tiberius	0:13
	BRAKER3	55:11
Osmia bicornis	Helixer	0:52
	ANNEVO	0:08
	Tiberius	0:10
	BRAKER3	25:45
Tribolium castaneum	Helixer	0:57
	ANNEVO	0:09
	Tiberius	0:09
	BRAKER3	40:03
Vanessa cardui	Helixer	1:29
	ANNEVO	0:13
	Tiberius	0:15
	BRAKER3	50:12
Zerene cesonia	Helixer	1:01
	ANNEVO	0:10
	Tiberius	0:10
	BRAKER3	27:41

Table 14: Runtime benchmark for Fungi.

Species	Tool	Runtime (h:mm)
Agaricus bisporus	Helixer	0:08
	ANNEVO	0:03
	Tiberius	0:02
	BRAKER3	1:38
Cryphonectria parasitica	Helixer	0:07
	ANNEVO	0:02
	Tiberius	0:01
	BRAKER3	1:10
Parastagonospora nodorum	Helixer	0:06
	ANNEVO	0:02
	Tiberius	0:01
	BRAKER3	1:30
Puccinia striiformis	Helixer	0:15
	ANNEVO	0:02
	Tiberius	0:02
	BRAKER3	2:11
Punctularia strigosozonata	Helixer	0:06
	ANNEVO	0:02
	Tiberius	0:01
	BRAKER3	1:55
Tilletiopsis washingtonensis	Helixer	0:03
	ANNEVO	0:01
	Tiberius	0:01
	BRAKER3	1:41

Table 15: Runtime benchmark for Mammalia.

Species	Tool	Runtime (h:mm)
Bos taurus	Helixer	10:53
	ANNEVO	1:24
	Tiberius	1:20
	BRAKER3	83:21
Delphinapterus leucas	Helixer	11:10
	ANNEVO	1:18
	Tiberius	1:16
	BRAKER3	76:40
Homo sapiens	Helixer	10:56
	ANNEVO	1:55
	Tiberius	1:27
	BRAKER3	71:22

Table 16: Runtime benchmark for Vertebrata.

Species	Tool	Runtime (h:mm)
Archocentrus centrarchus	Helixer	3:43
	ANNEVO	0:38
	Tiberius	0:36
	BRAKER3	40:26
Betta splendens	Helixer	1:39
	ANNEVO	0:17
	Tiberius	0:18
	BRAKER3	46:52
Gallus gallus	Helixer	4:21
	ANNEVO	0:44
	Tiberius	0:39
	BRAKER3	64:34
Pristiophorus japonicus	Helixer	21:24
	ANNEVO	4:16
	Tiberius	3:12
	BRAKER3	34:11
Takifugu rubripes	Helixer	1:29
	ANNEVO	0:12
	Tiberius	0:17
	BRAKER3	53:25
Zootoca vivipara	Helixer	6:06
	ANNEVO	0:52
	Tiberius	0:49
	BRAKER3	64:03

Table 17: Average runtime per clade and overall mean across all species, the number of species per average are indicated as n.

Clade	Tool	n	Runtime (h:mm)
Chlorophyta	Helixer	2	0:14
	Tiberius	2	0:03
	BRAKER3	2	17:29
Bacillariophyta	Tiberius	2	0:01
	BRAKER3	2	19:13
Mesangiospermae	Helixer	7	1:35
	ANNEVO	7	0:22
	Tiberius	7	0:17
	BRAKER3	7	20:26
Fungi	Helixer	6	0:08
	ANNEVO	6	0:02
	Tiberius	6	0:02
	BRAKER3	6	1:41
Insecta	Helixer	11	1:14
	ANNEVO	11	0:11
	Tiberius	11	0:12
	BRAKER3	11	45:42
Mammalia	Helixer	3	11:00
	ANNEVO	3	1:32
	Tiberius	3	1:21
	BRAKER3	3	77:08
Vertebrata	Helixer	6	6:27
	ANNEVO	6	1:10
	Tiberius	6	0:58
	BRAKER3	6	50:35
Overall	Helixer	33	2:57
	ANNEVO	33	0:30
	Tiberius	33	0:26
	BRAKER3	33	36:05

Clade	Species	Helixer	ANNEVO
Mesangiospermae	*Arabidopsis thaliana*	train+val	test
Mesangiospermae	*Brachypodium distachyon*	–	–
Mesangiospermae	*Eschscholzia californica*	–	–
Mesangiospermae	*Freycinetia multiflora*	–	–
Mesangiospermae	*Mimulus guttatus*	train+val	–
Mesangiospermae	*Sorghum bicolor*	train+val	val
Mesangiospermae	*Urochloa brizantha*	–	–
Fungi	*Agaricus bisporus*	train+val	test
Fungi	*Cryphonectria parasitica*	test	test
Fungi	*Parastagonospora nodorum*	train+val	test
Fungi	*Puccinia striiformis*	–	test
Fungi	*Punctularia strigosozonata*	train+val	test
Fungi	*Tilletiopsis washingtonensis*	train+val	test
Insecta	*Bombyx mori*	train+val	test
Insecta	*Cataglyphis hispanica*	–	train
Insecta	*Colias croceus*	test	test
Insecta	*Danaus plexippus*	test	–
Insecta	*Drosophila melanogaster*	train+val	test
Insecta	*Leptidea sinapis*	–	test
Insecta	*Nymphalis io*	–	–
Insecta	*Osmia bicornis*	train+val	–
Insecta	*Tribolium castaneum*	test	–
Insecta	*Vanessa cardui*	–	train
Insecta	*Zerene cesonia*	train+val	–
Mammalia	*Bos taurus*	test	test
Mammalia	*Delphinapterus leucas*	train+val	–
Mammalia	*Homo sapiens*	train+val	test
Vertebrata	*Archocentrus centrarchus*	train+val	test
Vertebrata	*Betta splendens*	train+val	test
Vertebrata	*Gallus gallus*	train+val	test
Vertebrata	*Pristiophorus japonicus*	train+val	–
Vertebrata	*Takifugu rubripes*	train+val	test
Vertebrata	*Zootoca vivipara*	test	test

Table 18: Usage of Tiberius test species during the training of the Helixer and ANNEVO models. None of the species were used to validate or train Tiberius.

Table 19: Average accuracy benchmark for all test species of each Tiberius model, compared to *ab initio* prediction of Helixer and ANNEVO, and evidence supported prediction of BRAKER3, where BRAKER3 used the proteomes of Tiberius' training species and short-read RNA-Seq.

Clade	Tool	n	Exon			Transcript			Gene		
			Sn	Pr	F1	Sn	Pr	F1	Sn	Pr	F1
Chlorophyta	Helixer	2	69.5	64.2	66.7	43.2	43.4	43.0	43.2	43.4	43.0
	Tiberius	2	66.3	67.0	66.6	44.7	44.9	44.7	44.7	44.9	44.7
	BRAKER3	2	51.2	66.8	57.6	42.0	46.8	44.3	42.1	55.7	47.9
Bacillariophyta	Tiberius	2	66.8	75.0	70.6	60.2	66.2	63.1	62.4	66.2	64.2
	BRAKER3	2	60.0	72.5	65.6	57.1	60.2	58.5	59.0	72.9	65.1
Mesangiospermae	Helixer	7	84.7	75.8	79.7	50.7	54.6	52.1	64.6	54.6	59.0
	ANNEVO	7	80.8	83.7	82.2	45.8	60.7	52.0	58.4	60.7	59.5
	Tiberius	7	83.9	89.5	86.6	56.7	69.9	62.3	72.5	69.9	71.0
	BRAKER3	7	80.6	92.4	86.0	59.2	73.3	65.2	73.7	79.0	76.0
Fungi	Helixer	6	64.9	53.8	58.7	32.2	26.6	29.0	32.2	26.6	29.0
	ANNEVO	6	66.1	62.8	64.3	35.6	35.3	35.4	35.6	35.3	35.4
	Tiberius	6	70.9	70.9	70.9	44.7	46.1	45.3	44.7	46.1	45.3
	BRAKER3	6	63.8	74.5	68.6	43.8	48.5	45.8	43.8	54.7	48.5
Insecta	Helixer	11	79.5	72.8	75.9	27.8	36.9	31.6	42.5	36.9	39.5
	ANNEVO	11	80.5	80.6	80.5	31.8	47.3	38.0	48.7	47.3	47.9
	Tiberius	11	83.2	85.7	84.4	44.6	50.5	47.1	68.1	50.5	57.6
	BRAKER3	11	82.1	92.1	86.7	56.8	67.1	61.3	76.4	74.7	75.4
Mammalia	Helixer	3	77.4	71.0	74.1	10.4	24.0	14.5	24.6	24.0	24.3
	ANNEVO	3	81.5	80.3	80.9	18.8	36.6	24.8	44.4	36.6	40.1
	Tiberius	3	83.4	90.7	86.9	27.3	59.5	37.4	64.6	59.5	61.9
	BRAKER3	3	69.3	93.7	79.6	33.9	66.2	44.8	68.9	72.6	70.4
Vertebrata	Helixer	6	78.7	68.2	72.9	12.8	21.0	15.7	23.3	21.0	22.1
	ANNEVO	6	81.6	80.0	80.7	21.7	36.6	26.8	38.9	36.6	37.6
	Tiberius	6	85.6	86.2	85.8	33.6	51.9	39.8	60.4	51.9	55.3
	BRAKER3	6	76.8	95.2	84.9	45.1	70.1	54.5	69.5	80.1	74.3
Overall	Helixer	33	77.6	69.0	72.9	29.1	34.7	31.0	40.2	34.7	37.2
	ANNEVO	33	78.2	77.9	78.0	32.4	45.1	37.2	46.2	45.1	45.5
	Tiberius	33	81.6	84.4	82.9	43.6	54.9	47.8	63.1	54.9	58.2
	BRAKER3	33	76.3	89.7	82.3	50.7	65.5	56.6	67.9	72.8	70.0

Table 20: Accuracy benchmark for the test species of the Tiberius Chlorophyta model compared to *ab initio* prediction of Helixer and evidence supported prediction of BRAKER3, where BRAKER3 used the proteomes of Tiberius' training species and short-read RNA-Seq.

Species	Tool	Exon			Transcript			Gene		
		Sn	Pr	F1	Sn	Pr	F1	Sn	Pr	F1
Bathycoccus prasinos	Helixer	52.9	52.4	52.6	52.7	59.2	55.8	52.7	59.2	55.8
	Tiberius	48.4	51.1	49.7	48.2	52.1	50.1	48.2	52.1	50.1
	BRAKER3	41.2	44.4	42.7	38.4	41.0	39.7	38.5	51.2	44.0
Edaphochlamys debaryana	Helixer	86.1	75.9	80.7	33.8	27.5	30.3	33.8	27.5	30.3
	Tiberius	84.3	82.8	83.5	41.2	37.6	39.3	41.2	37.6	39.3
	BRAKER3	61.1	89.3	72.6	45.6	52.6	48.9	45.7	60.1	51.9

Table 21: Accuracy benchmark for the test species of the Tiberius Bacillariophyta model compared to evidence supported prediction of BRAKER3, where BRAKER3 used the proteomes of Tiberius' training species and short-read RNA-Seq.

Species	Tool	Exon			Transcript			Gene		
		Sn	Pr	F1	Sn	Pr	F1	Sn	Pr	F1
Phaeodactylum tricornutum	Tiberius	58.7	70.0	63.9	57.7	62.7	60.1	57.7	62.7	60.1
	BRAKER3	59.8	67.3	63.3	60.3	58.2	59.2	60.4	69.5	64.6
Thalassiosira pseudonana	Tiberius	74.8	80.0	77.3	62.7	69.8	66.1	67.0	69.8	68.4
	BRAKER3	60.3	77.8	67.9	54.0	62.1	57.8	57.6	76.3	65.6

Table 22: Accuracy benchmark for the test species of the Tiberius Mesangiospermae model compared to *ab initio* prediction of Helixer and ANNEVO, and evidence supported prediction of BRAKER3, where BRAKER3 used the proteomes of Tiberius' training species and short-read RNA-Seq.

Species	Tool	Exon			Transcript			Gene		
		Sn	Pr	F1	Sn	Pr	F1	Sn	Pr	F1
Arabidopsis thaliana	Helixer	82.2	89.5	85.7	50.3	74.1	59.9	72.7	74.1	73.4
	ANNEVO	78.6	88.9	83.4	43.3	69.0	53.2	62.7	69.0	65.7
	Tiberius	82.4	95.8	88.6	53.7	85.7	66.0	77.7	85.7	81.5
	BRAKER3	80.9	94.7	87.3	55.9	77.9	65.1	78.6	83.9	81.2
Brachypodium distachyon	Helixer	74.6	77.6	76.1	38.0	53.6	44.5	49.6	53.6	51.5
	ANNEVO	69.8	85.1	76.7	32.9	60.1	42.5	42.9	60.1	50.1
	Tiberius	81.3	88.9	84.9	54.0	64.3	58.7	68.0	64.3	66.1
	BRAKER3	67.5	87.5	76.2	42.1	56.6	48.3	54.2	60.4	57.1
Eschscholzia californica	Helixer	82.7	75.1	78.7	43.1	52.4	47.3	61.0	52.4	56.4
	ANNEVO	78.6	84.8	81.6	39.0	61.7	47.8	55.2	61.7	58.3
	Tiberius	83.4	91.6	87.3	51.9	75.4	61.5	73.5	75.4	74.4
	BRAKER3	79.1	94.6	86.2	50.9	79.2	62.0	70.0	85.6	77.0
Freycinetia multiflora	Helixer	86.5	67.7	76.0	47.6	39.6	43.2	47.6	39.6	43.2
	ANNEVO	83.1	77.5	80.2	49.0	49.0	49.0	49.0	49.0	49.0
	Tiberius	81.9	85.2	83.5	48.8	56.4	52.3	65.2	56.4	60.5
	BRAKER3	81.5	92.1	86.5	67.3	69.6	68.4	67.3	79.9	73.1
Medicago truncatula	Helixer	85.7	73.7	79.2	54.1	49.9	51.9	63.8	49.9	56.0
	ANNEVO	82.9	83.9	83.4	50.8	60.8	55.4	59.9	60.8	60.3
	Tiberius	84.7	89.4	87.0	60.5	71.1	65.4	71.3	71.1	71.2
	BRAKER3	82.1	92.0	86.8	64.1	73.5	68.5	73.0	79.2	76.0
Mimulus guttatus	Helixer	89.2	84.4	86.7	60.8	66.4	63.5	74.3	66.4	70.1
	ANNEVO	85.2	84.9	85.0	52.7	62.4	57.1	64.3	62.4	63.3
	Tiberius	90.3	91.6	90.9	68.9	75.9	72.2	84.2	75.9	79.8
	BRAKER3	87.9	94.2	90.9	69.0	79.2	73.7	82.2	84.9	83.5
Urochloa brizantha	Helixer	87.6	62.2	72.7	62.0	43.1	50.9	62.0	43.1	50.9
	ANNEVO	82.3	79.3	80.8	54.4	56.2	55.3	54.4	56.2	55.3
	Tiberius	83.2	84.3	83.7	59.3	60.2	59.7	67.7	60.2	63.7
	BRAKER3	79.2	88.9	83.8	65.7	66.2	65.9	65.7	72.2	68.8

Table 23: Accuracy benchmark for the test species of the Tiberius Fungi model compared to *ab initio* prediction of Helixer and ANNEVO, and evidence supported prediction of BRAKER3, where BRAKER3 used the proteomes of Tiberius' training species and short-read RNA-Seq.

Species	Tool	Exon			Transcript			Gene		
		Sn	Pr	F1	Sn	Pr	F1	Sn	Pr	F1
Agaricus bisporus	Helixer	67.6	57.9	62.4	23.1	18.7	20.7	23.1	18.7	20.7
	ANNEVO	74.2	68.4	71.2	32.7	31.1	31.9	32.7	31.1	31.9
	Tiberius	79.6	77.5	78.5	44.0	44.3	44.1	44.0	44.3	44.1
	BRAKER3	74.6	78.2	76.4	45.7	45.4	45.5	45.7	52.4	48.8
Cryphonectria parasitica	Helixer	59.9	52.3	55.8	39.7	32.1	35.5	39.7	32.1	35.5
	ANNEVO	59.3	58.1	58.7	39.8	36.3	38.0	39.8	36.3	38.0
	Tiberius	62.3	65.6	63.9	45.2	43.6	44.4	45.2	43.6	44.4
	BRAKER3	59.8	65.6	62.6	45.5	41.9	43.6	45.5	47.9	46.7
Parastagonospora nodorum	Helixer	37.5	38.0	37.7	22.3	24.2	23.2	22.3	24.2	23.2
	ANNEVO	43.0	47.0	44.9	27.9	32.5	30.0	27.9	32.5	30.0
	Tiberius	45.6	53.7	49.3	32.0	38.8	35.1	32.0	38.8	35.1
	BRAKER3	38.6	54.3	45.1	29.4	38.3	33.3	29.4	42.4	34.7
Puccinia striiformis	Helixer	66.1	43.2	52.3	28.5	19.4	23.1	28.5	19.4	23.1
	ANNEVO	58.6	54.0	56.2	24.9	24.2	24.5	24.9	24.2	24.5
	Tiberius	67.9	66.3	67.1	38.4	37.3	37.8	38.4	37.3	37.8
	BRAKER3	64.5	85.1	73.4	47.9	59.3	53.0	48.0	66.7	55.8
Punctularia strigosozonata	Helixer	74.7	61.1	67.2	30.8	24.6	27.4	30.8	24.6	27.4
	ANNEVO	76.9	68.2	72.3	35.0	31.5	33.2	35.0	31.5	33.2
	Tiberius	82.3	77.5	79.8	47.3	46.0	46.6	47.4	46.0	46.7
	BRAKER3	72.4	78.5	75.3	45.5	46.3	45.9	45.5	52.2	48.6
Tilletiopsis washingtonensis	Helixer	83.4	70.6	76.5	48.9	40.6	44.4	48.9	40.6	44.4
	ANNEVO	84.7	80.8	82.7	53.0	56.2	54.6	53.0	56.2	54.6
	Tiberius	87.9	85.0	86.4	61.3	66.4	63.7	61.3	66.4	63.7
	BRAKER3	72.8	85.3	78.6	48.5	59.7	53.5	48.5	66.6	56.1

Table 24: Accuracy benchmark for the test species of the Tiberius Insecta model compared to *ab initio* prediction of Helixer and ANNEVO, and evidence supported prediction of BRAKER3, where BRAKER3 used the proteomes of Tiberius' training species and short-read RNA-Seq.

Species	Tool	Exon			Transcript			Gene		
		Sn	Pr	F1	Sn	Pr	F1	Sn	Pr	F1
Bombyx mori	Helixer	80.1	73.0	76.4	20.3	30.9	24.5	34.7	30.9	32.7
	ANNEVO	80.2	80.0	80.1	24.2	40.5	30.3	41.2	40.5	40.8
	Tiberius	77.8	85.6	81.5	35.4	41.9	38.4	60.2	41.9	49.4
	BRAKER3	81.7	94.2	87.5	48.7	71.7	58.0	73.1	79.7	76.3
Cataglyphis hispanica	Helixer	81.5	80.9	81.2	31.0	45.4	36.8	51.8	45.4	48.4
	ANNEVO	80.8	83.5	82.1	31.2	50.5	38.6	52.1	50.5	51.3
	Tiberius	84.1	86.0	85.0	43.3	47.8	45.4	72.4	47.8	57.6
	BRAKER3	85.4	88.8	87.1	57.9	56.1	57.0	81.1	60.3	69.2
Colias croceus	Helixer	85.0	75.1	79.7	30.5	35.5	32.8	42.6	35.5	38.7
	ANNEVO	83.6	82.9	83.2	33.8	47.3	39.4	47.2	47.3	47.2
	Tiberius	87.2	90.6	88.9	49.6	60.3	54.4	69.3	60.3	64.5
	BRAKER3	82.8	93.2	87.7	59.4	67.9	63.4	73.8	77.6	75.7
Danaus plexippus	Helixer	84.8	79.5	82.1	32.4	41.9	36.5	48.5	41.9	45.0
	ANNEVO	83.7	83.9	83.8	34.3	50.7	40.9	51.4	50.7	51.0
	Tiberius	87.5	91.5	89.5	49.4	62.1	55.0	73.9	62.1	67.5
	BRAKER3	86.7	93.5	90.0	61.8	66.9	64.2	80.8	74.4	77.5
Drosophila melanogaster	Helixer	79.2	84.1	81.6	43.1	66.0	52.1	68.9	66.0	67.4
	ANNEVO	77.5	86.0	81.5	41.8	68.5	51.9	66.7	68.5	67.6
	Tiberius	81.1	90.7	85.6	49.8	69.5	58.0	79.6	69.5	74.2
	BRAKER3	83.8	94.9	89.0	60.4	80.6	69.1	85.7	89.6	87.6
Leptidea sinapis	Helixer	78.9	59.6	67.9	18.3	21.4	19.7	28.9	21.4	24.6
	ANNEVO	80.6	70.2	75.0	25.5	33.8	29.1	40.3	33.8	36.8
	Tiberius	80.3	74.3	77.2	38.2	30.9	34.2	60.1	30.9	40.8
	BRAKER3	75.1	86.5	80.4	48.0	61.8	54.0	68.5	68.0	68.2
Nymphalis io	Helixer	84.2	74.3	78.9	27.8	35.5	31.2	42.9	35.5	38.9
	ANNEVO	84.5	80.3	82.3	33.7	46.6	39.1	52.1	46.6	49.2
	Tiberius	87.2	86.9	87.0	46.2	50.1	48.1	71.3	50.1	58.8
	BRAKER3	85.2	94.5	89.6	59.3	71.9	65.0	79.9	81.3	80.6
Osmia bicornis	Helixer	77.2	77.7	77.4	26.4	41.7	32.3	43.9	41.7	42.8
	ANNEVO	75.8	81.2	78.4	26.7	47.1	34.1	44.4	47.1	45.7
	Tiberius	80.6	79.2	79.9	40.2	36.4	38.2	66.8	36.4	47.1
	BRAKER3	81.2	89.7	85.2	51.3	58.1	54.5	72.5	64.6	68.3
Tribolium castaneum	Helixer	53.3	48.1	50.6	13.2	15.7	14.3	20.9	15.7	17.9
	ANNEVO	68.5	74.2	71.2	25.2	39.0	30.6	39.8	39.0	39.4
	Tiberius	74.6	81.8	78.0	38.3	46.7	42.1	60.4	46.7	52.7
	BRAKER3	83.2	90.7	86.8	57.7	67.4	62.2	80.2	74.6	77.3
Vanessa cardui	Helixer	85.2	75.0	79.8	31.9	37.8	34.6	43.7	37.8	40.5
	ANNEVO	85.4	84.1	84.7	38.4	51.8	44.1	52.5	51.8	52.1
	Tiberius	88.0	87.1	87.5	50.8	50.5	50.6	69.5	50.5	58.5
	BRAKER3	84.0	93.8	88.6	60.3	68.9	64.3	73.6	78.3	75.9
Zerene cesonia	Helixer	85.1	73.9	79.1	30.4	34.1	32.1	41.0	34.1	37.2
	ANNEVO	84.5	80.8	82.6	35.2	44.7	39.4	47.6	44.7	46.1
	Tiberius	87.3	89.3	88.3	48.9	59.0	53.5	66.0	59.0	62.3
	BRAKER3	73.8	93.1	82.3	59.6	66.6	62.9	71.5	73.7	72.6

Table 25: Accuracy benchmark for the test species of the Tiberius Mammalia model compared to *ab initio* prediction of Helixer and ANNEVO, and evidence supported prediction of BRAKER3, where BRAKER3 used the proteomes of Tiberius' training species and short-read RNA-Seq.

Species	Tool	Exon			Transcript			Gene		
		Sn	Pr	F1	Sn	Pr	F1	Sn	Pr	F1
Bos taurus	Helixer	76.2	70.7	73.3	10.2	24.0	14.3	23.1	24.0	23.5
	ANNEVO	81.3	82.8	82.0	20.3	41.4	27.2	46.2	41.4	43.7
	Tiberius	82.5	90.2	86.2	27.3	57.8	37.1	62.2	57.8	59.9
	BRAKER3	67.2	94.9	78.7	32.9	71.3	45.0	64.4	77.0	70.1
Delphinapterus leucas	Helixer	77.3	72.5	74.8	10.6	23.0	14.5	23.3	23.0	23.1
	ANNEVO	80.5	77.5	79.0	17.8	30.0	22.3	39.3	30.0	34.0
	Tiberius	82.6	91.8	87.0	27.3	59.4	37.4	60.3	59.4	59.8
	BRAKER3	66.6	94.1	78.0	34.6	65.8	45.4	65.8	72.4	68.9
Homo sapiens	Helixer	78.8	69.8	74.0	10.5	25.0	14.8	27.4	25.0	26.1
	ANNEVO	82.8	80.5	81.6	18.3	38.4	24.8	47.7	38.4	42.5
	Tiberius	85.0	90.2	87.5	27.4	61.2	37.9	71.4	61.2	65.9
	BRAKER3	74.0	92.0	82.0	34.3	61.5	44.0	76.5	68.4	72.2

Table 26: Accuracy benchmark for the non-mammalian test species of the Tiberius Vertebrata model compared to *ab initio* prediction of Helixer and ANNEVO, and evidence supported prediction of BRAKER3, where BRAKER3 used the proteomes of Tiberius' training species and short-read RNA-Seq.

Species	Tool	Exon			Transcript			Gene		
		Sn	Pr	F1	Sn	Pr	F1	Sn	Pr	F1
Archocentrus centrarchus	Helixer	82.2	68.7	74.8	16.9	20.7	18.6	24.2	20.7	22.3
	ANNEVO	84.1	80.7	82.4	30.0	40.6	34.5	43.0	40.6	41.8
	Tiberius	88.2	86.1	87.1	44.9	53.6	48.9	64.3	53.6	58.5
	BRAKER3	76.9	94.3	84.7	55.4	69.7	61.7	69.4	79.4	74.1
Betta splendens	Helixer	82.5	79.0	80.7	16.9	30.0	21.6	32.3	30.0	31.1
	ANNEVO	84.2	86.5	85.3	24.9	47.0	32.6	47.6	47.0	47.3
	Tiberius	88.8	93.5	91.1	37.8	68.3	48.7	72.2	68.3	70.2
	BRAKER3	84.5	96.4	90.1	48.3	73.8	58.4	77.9	84.6	81.1
Gallus gallus	Helixer	78.1	72.2	75.0	10.2	25.7	14.6	26.3	25.7	26.0
	ANNEVO	77.3	84.5	80.7	14.6	40.3	21.4	37.6	40.3	38.9
	Tiberius	82.1	89.9	85.8	23.4	57.9	33.3	60.3	57.9	59.1
	BRAKER3	76.7	96.2	85.4	32.9	73.6	45.5	69.9	82.7	75.8
Pristiophorus japonicus	Helixer	67.6	47.0	55.4	5.8	6.4	6.1	9.6	6.4	7.7
	ANNEVO	77.9	66.2	71.6	15.3	17.5	16.3	25.4	17.5	20.7
	Tiberius	80.3	67.5	73.3	23.5	17.0	19.7	38.9	17.0	23.7
	BRAKER3	62.3	94.3	75.0	36.8	65.4	47.1	52.7	73.3	61.3
Takifugu rubripes	Helixer	80.9	75.7	78.2	15.1	24.8	18.8	27.2	24.8	25.9
	ANNEVO	82.9	83.4	83.1	23.7	41.4	30.1	42.7	41.4	42.0
	Tiberius	87.1	91.2	89.1	37.0	61.8	46.3	66.6	61.8	64.1
	BRAKER3	80.3	96.2	87.5	48.3	72.7	58.0	75.1	83.8	79.2
Zootoca vivipara	Helixer	81.2	66.4	73.1	11.8	18.6	14.4	20.3	18.6	19.4
	ANNEVO	83.4	78.8	81.0	21.4	33.0	26.0	36.9	33.0	34.8
	Tiberius	87.3	89.0	88.1	34.8	52.5	41.9	60.0	52.5	56.0
	BRAKER3	80.3	93.8	86.5	49.1	65.7	56.2	71.7	77.0	74.3

Table 27: Accuracy comparison between the Tiberius version v1.1.8 and v2.0.0 with reimplemnted core. The table shows the average accuracy across all test species for each Tiberius model.

Clade	Tool	n	Exon			Transcript			Gene		
			Sn	Pr	F1	Sn	Pr	F1	Sn	Pr	F1
Chlorophyta	Tiberius v1.1.8	2	66.2	67.0	66.6	44.6	44.9	44.7	44.6	44.9	44.7
	Tiberius v2.0.0	2	66.3	67.0	66.6	44.7	44.9	44.7	44.7	44.9	44.7
Bacillariophyta	Tiberius v1.1.8	2	66.7	75.1	70.6	60.2	66.3	63.1	62.2	66.3	64.2
	Tiberius v2.0.0	2	66.8	75.0	70.6	60.2	66.2	63.1	62.4	66.2	64.2
Mesangiospermae	Tiberius v1.1.8	7	82.1	90.8	86.1	56.6	71.3	62.3	66.5	71.3	68.6
	Tiberius v2.0.0	7	82.1	90.8	86.1	56.6	71.2	62.2	66.5	71.2	68.6
Fungi	Tiberius v1.1.8	6	70.8	71.0	70.8	44.7	46.2	45.3	44.7	46.2	45.3
	Tiberius v2.0.0	6	70.9	70.9	70.9	44.7	46.1	45.3	44.7	46.1	45.3
Insecta	Tiberius v1.1.8	11	83.5	86.1	84.7	44.9	51.6	47.8	68.7	51.6	58.5
	Tiberius v2.0.0	11	83.2	85.7	84.4	44.6	50.5	47.1	68.1	50.5	57.6
Mammalia	Tiberius v1.1.8	3	82.4	90.8	86.4	27.3	59.7	37.4	64.5	59.7	62.0
	Tiberius v2.0.0	3	83.4	90.7	86.9	27.3	59.5	37.4	64.6	59.5	61.9
Vertebrata	Tiberius v1.1.8	6	85.3	86.2	85.6	33.5	51.9	39.8	60.3	51.9	55.3
	Tiberius v2.0.0	6	85.6	86.2	85.8	33.6	51.9	39.8	60.4	51.9	55.3
Overall	Tiberius v1.1.8	37	79.5	83.3	81.3	44.6	55.6	48.6	61.0	55.6	57.6
	Tiberius v2.0.0	37	79.6	83.2	81.2	44.5	55.2	48.4	60.9	55.2	57.3

Table 28: Runtime comparison between the Tiberius v1.1.8 and and v2.0.0 with reimplemented core.

Clade	Tool	n	Runtime (h:mm)
Chlorophyta	Tiberius v.1.1.8	2	0:04
	Tiberius v2.0.0	2	0:03
Bacillariophyta	Tiberius v.1.1.8	2	0:02
	Tiberius v2.0.0	2	0:01
Mesangiospermae	Tiberius v.1.1.8	7	0:32
	Tiberius v2.0.0	7	0:21
Fungi	Tiberius v.1.1.8	6	0:02
	Tiberius v2.0.0	6	0:02
Insecta	Tiberius v.1.1.8	11	0:18
	Tiberius v2.0.0	11	0:12
Mammalia	Tiberius v.1.1.8	3	1:58
	Tiberius v2.0.0	3	1:20
Vertebrata	Tiberius v.1.1.8	6	1:22
	Tiberius v2.0.0	6	0:58
Overall	Tiberius v.1.1.8	37	0:35
	Tiberius v2.0.0	37	0:24

Table 29: The names of the models used for Tiberius, ANNEVO and Helixer for the benchmark on the test species for each clade.

Clade	Tiberius model	ANNEVO model	Helixer model
Chlorophyta	chlorophyta		land_plant
Bacillariophyta	diatoms_unmasked		
Mesangiospermae	angiosperms	ANNEVO_Embryophyta.pt	land_plant
Fungi	fungi	ANNEVO_Fungi.pt	fungi
Insecta	insecta_unmasked_v2	ANNEVO_Invertebrate.pt	invertebrate
Mammalia	mammalia_nosofttmasking_v2	ANNEVO_Mammalia2.pt	vertebrate
Vertebrata	vertebrate	ANNEVO_Vertebrate_other.pt	vertebrate

Model / Clade	GPU	Phase 2 (HMM training)
Chlorophyta	A100 (80 GB)	Yes
Bacillariophyta	A100 (80 GB)	Yes
Mesangiospermae	A100 (40 GB)	No
Insecta	A100 (80 GB)	Yes
Fungi	A100 (80 GB)	Yes
Mammalia	A100 (80 GB)	Yes
Vertebrata	4x NVIDIA H100 (64 GB VRAM each)	Yes

Table 30: Overview of the GPU hardware used for training each model and whether training included a second phase with the HMM layer.

Model / Clade	CNN units	LSTM units	Params (M)
Chlorophyta	128	744	8.0
Bacillariophyta	128	744	8.0
Mesangiospermae	128	744	8.0
Insecta	128	372	2.5
Fungi	128	744	8.0
Vertebrata	256	744	9.8

Table 31: Model sizes for all trained clade-specific Tiberius models. The overall architecture is identical to the original mammalian Tiberius model, consisting of three CNN followed by two biLSTM layers. Only layer sizes (CNN units/channels and LSTM units) were varied between the models, resulting in different total parameter counts.
